# Serial-femtosecond crystallography reveals how a phytochrome variant couples chromophore and protein structural changes

**DOI:** 10.1126/sciadv.adp2665

**Published:** 2025-05-28

**Authors:** Luisa Sauthof, Michal Szczepek, Andrea Schmidt, Asmit Bhowmick, Medhanjali Dasgupta, Megan J. Mackintosh, Sheraz Gul, Franklin D. Fuller, Ruchira Chatterjee, Iris D. Young, Norbert Michael, Nicolas Andreas Heyder, Brian Bauer, Anja Koch, Isabel Bogacz, In-Sik Kim, Philipp S. Simon, Agata Butryn, Pierre Aller, Volha U. Chukhutsina, James M. Baxter, Christopher D. M. Hutchison, Dorothee Liebschner, Billy Poon, Nicholas K. Sauter, Mitchell D. Miller, George N. Phillips, Roberto Alonso-Mori, Mark S. Hunter, Alexander Batyuk, Shigeki Owada, Kensuke Tono, Rie Tanaka, Jasper J. van Thor, Norbert Krauß, Tilman Lamparter, Aaron S. Brewster, Igor Schapiro, Allen M. Orville, Vittal K. Yachandra, Junko Yano, Peter Hildebrandt, Jan F. Kern, Patrick Scheerer

**Affiliations:** ^1^ Charité–Universitätsmedizin Berlin, corporate member of Freie Universität Berlin and Humboldt-Universität zu Berlin, Institute of Medical Physics and Biophysics, Group Structural Biology of Cellular Signaling, Charitéplatz 1, D-10117, Berlin, Germany.; ^2^ Molecular Biophysics and Integrated Bioimaging Division, Lawrence Berkeley National Laboratory, 1 Cyclotron Road, Berkeley, CA 94720, USA.; ^3^ Fritz Haber Center for Molecular Dynamics Research, Institute of Chemistry, The Hebrew University of Jerusalem, Jerusalem 9190401, Israel.; ^4^ Linac Coherent Light Source (LCLS), SLAC National Accelerator Laboratory, 2575 Sand Hill Road, MS103, Menlo Park, CA 94025, USA.; ^5^ Technische Universität Berlin, Institut für Chemie, Sekr. PC 14, Straße des 17. Juni 135, Berlin D-10623, Germany.; ^6^ Diamond Light Source Ltd., Harwell Science and Innovation Campus, Didcot, Oxfordshire, OX11 0DE, UK.; ^7^ Research Complex at Harwell, Harwell Science and Innovation Campus, Didcot, Oxfordshire, OX11 0FA, UK.; ^8^ Imperial College London, Life Sciences Department, South Kensington Campus, London SW7 2AZ, UK.; ^9^ Rice University, Department of Biosciences, 6100 Main Street, Houston, TX 77005, USA.; ^10^ Japan Synchrotron Radiation Research Institute, Kouto 1-1-1, Sayo, Hyogo 679-5198, Japan.; ^11^ RIKEN SPring-8 Center, 1-1-1 Kouto, Sayo, Hyogo, 679-5148, Japan.; ^12^ Department of Cell Biology, Graduate School of Medicine, Kyoto University, Yoshidakonoecho, Sakyo-ku, Kyoto, 606-8501, Japan.; ^13^ Karlsruhe Institute of Technology (KIT), Joseph Gottlieb Kölreuter Institute of Plant Science (JKIP), Fritz Haber Weg 4, D-76131 Karlsruhe, Germany.; ^14^ Department of Physics, Technical University Dortmund, Dortmund, Germany.; ^15^ Research Center Chemical Sciences and Sustainability, University Alliance Ruhr, 44801 Bochum, Germany.

## Abstract

The photoreaction and commensurate structural changes of a chromophore within biological photoreceptors elicit conformational transitions of the protein promoting the switch between deactivated and activated states. We investigated how this coupling is achieved in a bacterial phytochrome variant, Agp2-PAiRFP2. Contrary to classical protein crystallography, which only allows probing (cryo-trapped) stable states, we have used time-resolved serial femtosecond x-ray crystallography (tr-SFX) and pump-probe techniques with various illumination and delay times with respect to photoexcitation of the parent Pfr state. Thus, structural data for seven time frames were sorted into groups of molecular events along the reaction coordinate. They range from chromophore isomerization to the formation of Meta-F, the intermediate that precedes the functional relevant secondary structure transition of the tongue. Structural data for the early events were used to calculate the photoisomerization pathway to complement the experimental data. Late events allow identifying the molecular switch that is linked to the intramolecular proton transfer as a prerequisite for the following structural transitions.

## Teaser

Serial-femtosecond x-ray crystallography reveals coupling of chromophore and protein structural changes in a phytochrome.

## INTRODUCTION

**Fig. 1.**
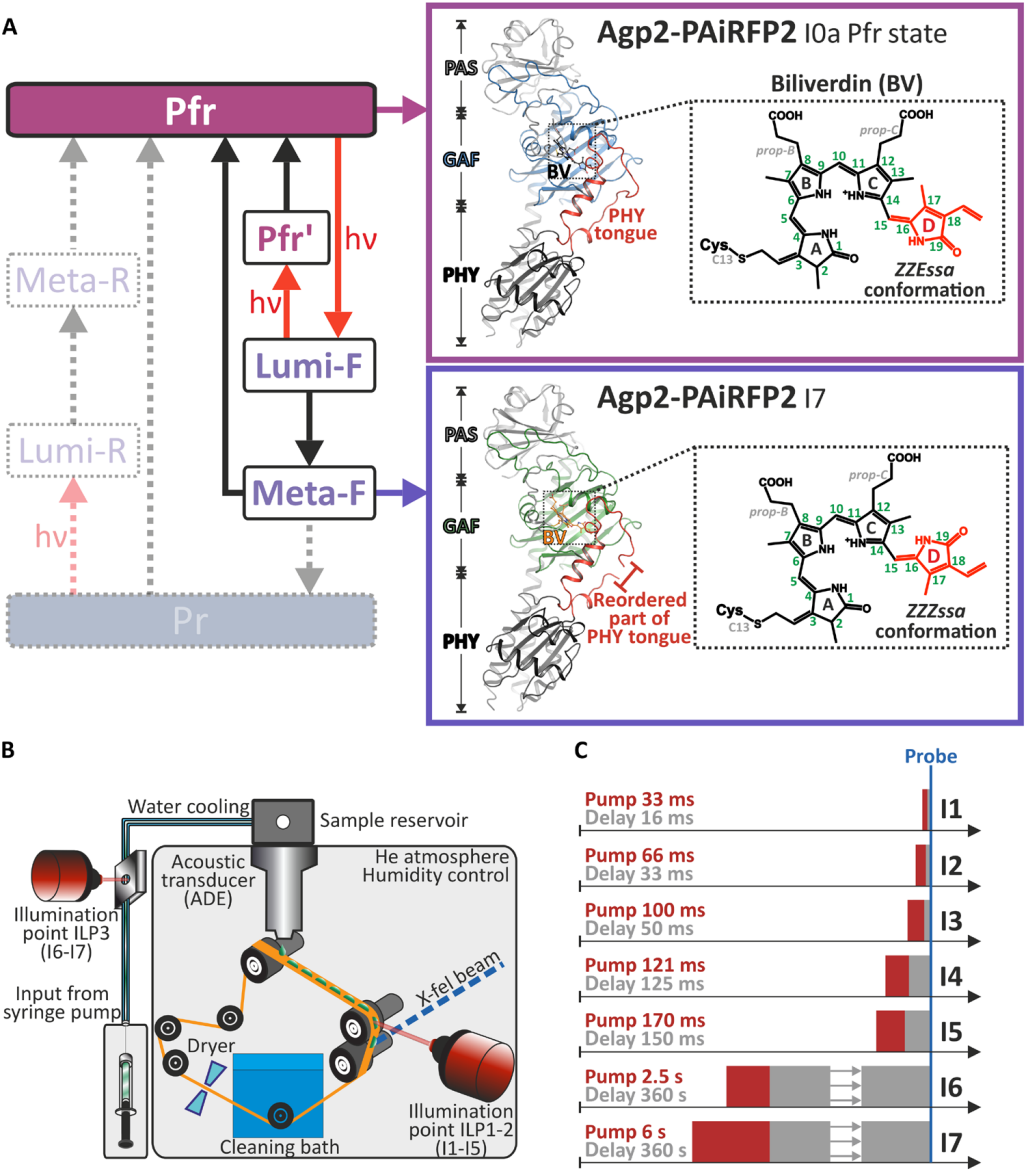
Overview of the light-induced reaction sequence of Agp2 and the scheme of the pump-probe XFEL measurements. (**A**) Left: Simplified scheme of the photocycle of the bathy phytochrome Agp2. Red and black arrows refer to photochemical and thermal reactions, respectively. Dotted arrows indicate reactions that take place only in Agp2-WT but not in Agp2-PAiRFP2. The gray-shaded states (Meta-R and Lumi-R) only occur in Agp2-WT. Right: The structure of Agp2-PAiRFP2 in Pfr (corresponding to I0a of the present XFEL measurements; PDB ID 8RJM, top) and its final photoinduced reaction product Meta-F (corresponding to I7 of the present XFEL measurements; PDB ID 8RJU, bottom), together with the respective structural formula of the chromophores and attachment to Cys^13^. The red-violet and deep-violet colored boxes represent the states with a *ZZEssa* and *ZZZssa* configuration of BV. (**B**) Representation of the drop-on-demand setup used in the pump-probe XFEL experiments (*40*). They were performed at room temperature in the dark to avoid unwanted photochemical processes, using 785- and 770-nm pump light-emitting diodes (LEDs) for the time frames I1 to I3, I6, I7, and I4 and I5, respectively. Two pump-probe schemes were used. For measurements on the millisecond timescale, Agp2-PAiRFP2 crystals were irradiated on the conveyor belt (illumination point ILP1-2). Different illumination and relaxation times were obtained by adjusting the belt speed and illumination point (see also the Supplementary Materials). A humidity control was installed to minimize dehydration effects on the conveyor belt. For longer time experiments, the crystals were irradiated inside the capillary to prevent dehydration (illumination point ILP3). Temperature control was ensured during sample injection and capillary perfusion. (**C**) Overview of the datasets collected at the XFEL facilities LCLS (Stanford, USA) and SACLA (Kouto, Japan) under different illumination and relaxation schemes (red: pump time; gray: delay time). The blue vertical line indicates the probe event with the x-ray pulses.

Organisms use light to regulate essential physiological processes such as the gene expression, circadian rhythms, motility, or vision (*1*, *2*). Light sensing is initiated by the photoreaction of a chromophore embedded in a photoreceptor protein. In plants, bacteria, and fungi, phytochrome sensory photoreceptors carrying a bilin chromophore switch between inactive and active states, denoted as Pr and Pfr, respectively (*3*). All phytochromes share a common photo-sensory core module (PCM) consisting of a PAS (N-terminal Per Arnt Sim), GAF (cGMP-specific phosphodiesterases, adenylyl cyclases, and FhlA), and PHY (phytochrome-specific) domain (Fig. 1A) (*4*–*6*). The PCM is linked to the output module which frequently includes a histidine kinase (*3*, *7*–*10*). In addition to different bilin-type chromophores and different covalent binding sites (*3*), in all phytochromes, the parent states Pr and Pfr are interconverted via photoconversion pathways with a similar mechanistic pattern, including chromophore isomerization, proton transfer, and secondary structure changes, although details are different in the various reaction routes (*11*, *12*). In prototypical phytochromes, Pr is the thermodynamically stable state which is also recovered from Pfr within a dark reaction. However, the relative stability of Pr and Pfr is reversed in some bacterial phytochromes, which are called bathy phytochromes. These phytochromes undergo Pr-to-Pfr dark conversion, and Pfr is the thermodynamically stable state (*13*–*15*).

For the bathy phytochrome Agp2 from *Agrobacterium fabrum*, the Pfr-to-Pr photoconversion has been studied extensively (Fig. 1A) (*12*, *16*–*23*). However, only limited structural studies under cryogenic conditions were carried out so far, leaving the precise sequence of structural events unclear at the molecular level. Starting from the Pfr state, the biliverdin (BV) chromophore undergoes a *E*-to-*Z* photoisomerization of the double bond at the methine bridge between the pyrrole rings *C* and *D*. This *E*-to-Z conversion is accompanied by restructuring of the surrounding hydrogen bonding network in the first spectroscopically defined intermediate state Lumi-F and followed by a relaxation of the chromophore and the chromophore binding pocket (CBP) to yield the Meta-F state. This state constitutes a branching point, as it can either decay directly back to Pfr or to Pr accompanied by an α helix–to–β sheet transition of the “tongue,” a peptide segment of the PHY domain that is close to the CBP (*22*, *24*, *25*). This restructuring of the tongue, probably universal in phytochromes (*26*, *27*), is coupled to further changes of the tertiary structure and lastly leads to the (de)activation of the output module. Meta-F is, therefore, the key intermediate in (bathy) phytochromes for translating light into physiological function.

In the Agp2-PAiRFP2 variant, optimized for fluorescence in view of potential optogenetic applications (*28*), the Meta-F state represents the final photoproduct of Pfr photoconversion since its decay to Pr and thus the helix-sheet transition of the tongue are blocked (Fig. 1A). Furthermore, in this variant, the dark reversion from Meta-F to Pfr is very slow, and consequently, the long lifetime of this intermediate allowed a rare opportunity for combined and thorough spectroscopic and structural characterization after flash cooling and diffraction data collection at 100 K (*19*). Thus, various key residues responsible for structural changes in the CBP were identified, including structural reorientations of Tyr^165^ and Phe^192^ in the vicinity of the isomerization site of BV and a displacement of Gln^190^ toward the conserved Trp^440^ in the tongue (*19*). It is hypothesized that this steric interaction is essential for the secondary structure transition of the tongue in wild-type Agp2 (Agp2-WT). In addition, the conserved Arg^242^ and Arg^211^ as well as Tyr^205^ are rearranged during the relaxation of the propionate side chains *B* and *C* (*prop-B* and *prop-C*) of BV which, in Agp2-WT, is presumably linked to the deprotonation of *prop-C*. Last, the covalent attachment of Cys^13^ to the ring *A* of BV changes from the β- to the α-facial orientation, followed by the remodeling of the N terminus of the photoreceptor (*19*).

Spectroscopic studies suggest that the photoinduced structural changes of Agp2-PAiRFP2 including those observed in the Meta-F substates are very similar to Agp2-WT, except for the proton transfer from *prop-C* to His^278^, which is a prerequisite for the α helix–to–β sheet transition of the tongue, thus opening the gate for the decay to Pr (*20*). In Meta-F of Agp2-PAiRFP2, this gate remains closed, and only a small part of the tongue was found to be structurally destabilized. The Agp2-PAiRFP2 variant is therefore ideally suited to study the light-induced reaction cascade of this photoreceptor up to the point of coupling of CBP structural changes with the functionally crucial transformation of the tongue.

Structural data by synchrotron (SR) single crystal cryo-crystallography and cryo-spectroscopy provide some insights into the phytochrome photocycle, while the detailed sequence of events and their temporal correlations remain elusive. Moreover, SR-based crystallography studies fully correlated with electronic absorption and/or resonance Raman spectroscopies using related phytochromes indicate that the family of proteins exhibit extreme radiation sensitivity (*29*, *30*), wherein the electronic structure changes with x-ray exposure, but the atomic structure is fixed by the cryogenic conditions. Thus, time-resolved crystallography to determine structural changes at physiological temperatures is required, which can be readily achieved by serial femtosecond crystallography (SFX) using the x-ray pulses generated by a free electron laser (XFEL). This technique has recently been used to probe individual states of prototypical phytochromes during the Pr-to-Pfr photoconversion, generated after an ultrashort and a millisecond delay time after the actinic light pulse (*31*, *32*). In the present work, we used a pump-probe time-resolved XFEL approach to analyze eight crystal structures of the Pfr-to-Pr photoconversion of Agp2-PAiRFP2 at ambient temperature and covering the structural changes in CBP up to the coupling with functional conformational changes of the protein (Fig. 1 and table S1).

## RESULTS

### The dark-adapted Pfr ground state of Agp2-PAiRFP2

**Fig. 2.**
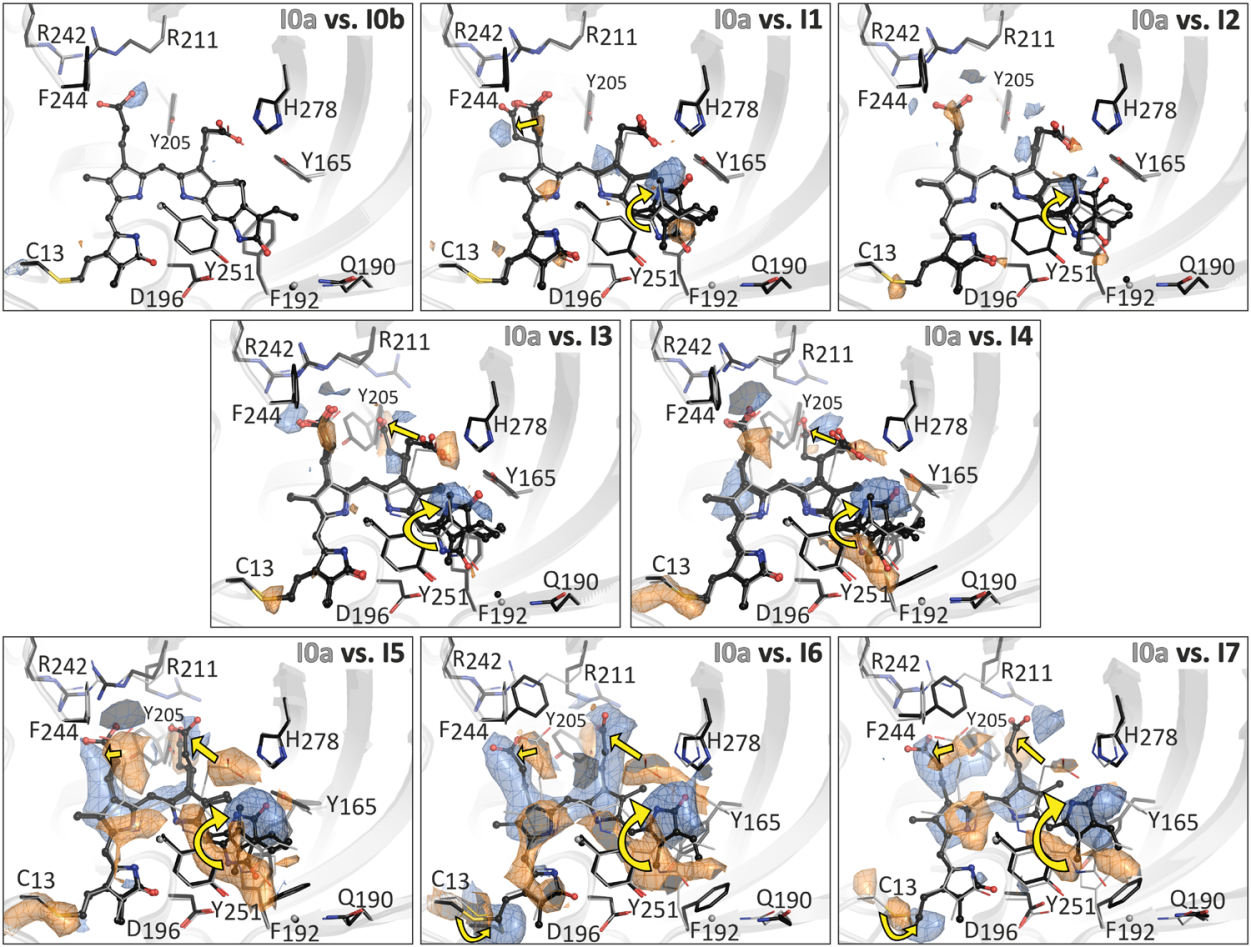
Time-resolved structural changes of MolA of Agp2-PAiRFP2 visualized from the *F*o^illuminated-state^ − *F*o^dark-adapted state^ electron density difference map. The *F*o^illuminated-state^ − *F*o^Pfr-state^ electron density difference map of Agp2-PAiRFP2 shows structural changes after photoisomerization of the chromophore. Blue colored electron density (contoured at +3 σ) indicates novel features obtained at different pump and delay times, whereas orange colored electron density (−3 σ) represent features of the initial dark state Pfr. The *F*o-*F*o electron density maps are contoured at the 3.0 σ level. The protein backbone is shown in cartoon representation. The BV and amino acid side chains of the CBP are represented as balls/sticks and sticks, respectively. In the Pfr state I0a, BV and amino acid side chains are colored in light gray. Structures of Pfr in I0b and all the time frames are shown in black. The pump-probe scheme related to the different time frames is described in Figs. 1 and 3.

The pump-probe experiments were carried out at two free electron laser sources, Linac Coherent Light Source (LCLS; Stanford, USA) and SPring-8 Angstrom Compact free electron LAser (SACLA; Kouto, Japan). As a starting point for the time-resolved structural analysis of Agp2-PAiRFP2, two room temperature datasets I0a (LCLS, 2.15-Å resolution) and I0b (SACLA, 2.20-Å resolution) of the dark-state Pfr were collected (Fig. 2, figs. S1 to S3, and tables S2 to S4). These room temperature structures are very similar to previous atomic models derived from cryogenic conditions as demonstrated by the root mean square deviation of 0.3 Å^2^ [relative to molecule A (Mol A)] for equivalent Cα atoms between the SR (*19*) and the SFX structures. The chromophore adopts the characteristic *ZZEssa* configuration and is α-facially bound to Cys^13^ (Fig. 2). The hydrogen bond network of the chromophore includes Tyr^165^, Asp^196^, Arg^211^, Tyr^251^, and His^278^. The backbone of the highly conserved Asp^196^ and the “pyrrole water” form hydrogen bonds with the N-H groups of the pyrrole rings *A*, *B*, and *C*. Several other water molecules mediate additional hydrogen bonds, extending the hydrogen bond network to Gln^190^, Tyr^205^, Arg^242^, Ser^245^, and Ser^260^. Last, the tongue of the PHY domain has a well-defined helical conformation. These structures are also very similar to the Pfr states of Agp2-WT and other bathy phytochromes (*33*).

### Time-resolved structural data of the photoactivation of Agp2-PAiRFP2

**Fig. 3.**
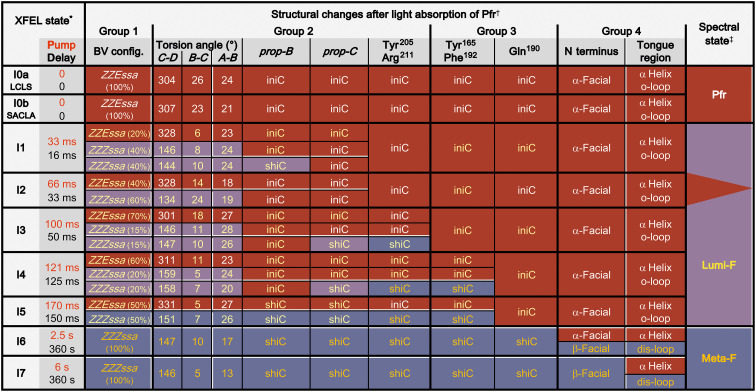
Structural changes of Agp2-PAiRFP2 crystals during the pump-probe XFEL experiments. XFEL states corresponding to the various time frames refer to the datasets obtained from LCLS (I0a, I1 to I3, I6, and I7) and SACLA (I0b, I4, and I5) with femtosecond x-ray pulses after the indicated pump (red font color) and delay time (black font color) as described in Fig. 1B. ^†^Coarse description of structural changes (for Mol A) during the XFEL experiments, gathered into molecular groups as described in the text. Group 1 describes the stereochemistry of the BV chromophore, whereby some models were refined with two—three configurations with different torsion (tilt) angles. “Initial” (iniC) and “shifted (shiC)” conformations refer to the initial Pfr state and the structural changes on the way to the final Meta-F state, respectively; the torsional (tilt) angles (only shown for Mol A and with values for single, double and triple conformations) of the BV methine bridges *C-D*, *B-C*, and *A-B* are expressed by the sum of the two dihedral angles (single and double dihedral bonds in BV) of the respective bridges; the α-helical part of the tongue remains. The initially ordered loop region (o-loop) of the tongue is partially disordered (dis-loop) in the final state; the N-terminal undergoes a transition from the α- to the β-facial orientation in the last step of the photoconversion. White font refers to the initial Pfr state, yellow font indicates excitation, and thus beginning structural rearrangements and orange font represent the final photoproduct. ^‡^Spectrally defined states, as identified by various optical spectroscopies (*12*, *16*–*23*), are taken from the reaction scheme in Fig. 1A. Here, we provide a tentative correlation between the distinct (spectroscopically defined) states and the XFEL states.

Seven time-resolved SFX datasets were collected to between 2.30- and 2.80-Å resolution at different time frames relative to photoexcitation that ranged from a 33-ms pump/16-ms delay time to a 6-s pump/360-s delay time (Figs. 1 and 3 and tables S1 to S6). The individual time frames (I1 to I7) do not correspond to discrete intermediates with energy minima on the potential hypersurface but reflect average structures of the system when probed in the respective time window. The pump-probe experiments allow us to dissect the photoactivation process and to follow the sequence of events at the structural level with reference to the parent state Pfr. Among the two molecules (Mol A and Mol B) in the crystals, we mainly focused on Mol A. Photoconversion in Mol B is most likely influenced by crystal packing effects, since the N terminus is blocked (figs. S3 to S5) (*19*). We have briefly explained the differences in Mol B in more detail in two figures in the Supplementary Materials (figs. S4 and S5). To facilitate the description, the structural changes are sorted into four groups, each comprising molecular events. These events occur on different but overlapping timescales and are directly related to spectroscopically distinct intermediates (Fig. 3).

### Group 1 of molecular events (<30 ms)—Chromophore isomerization

The first group of molecular events includes the isomerization of the C15─C16 bond at the methine bridge of BV, converting it from the *ZZEssa* in Pfr to the *ZZZssa* configuration in Lumi-F. The ring *D* rotation causes a change of hydrogen bonding partners, e.g., of the carbonyl group from a conserved water molecule to Tyr^165^. The present time resolution does not allow monitoring early events of the photoprocesses as captured in temperature-scan cryo-crystallography on the related bathy phytochrome (*34*). Instead, we observed the structurally relaxed photoisomerized chromophore. Isomerization was observed in all illuminated crystals albeit to different extents as determined from peaks in the various electron density maps (Fig. 2 and figs. S1, S2, and S6). After 33 ms of irradiation and a 16-ms delay time (I1), 80% of the chromophores were isomerized; however, the occupancy of the *ZZZssa* configuration decreased to as low as 30% at longer irradiation times (I2 and I3) before increasing again from I4 (121-ms irradiation and 125-ms delay time) onward to reach almost 100% in I6. These observations suggest that during the early time frames, a part of the isomerized chromophore is converted back from Lumi-F to Pfr, which agrees with spectroscopic data indicating a photoisomerization of the late Lumi-F (Fig. 1) (*35*).

**Fig. 4.**
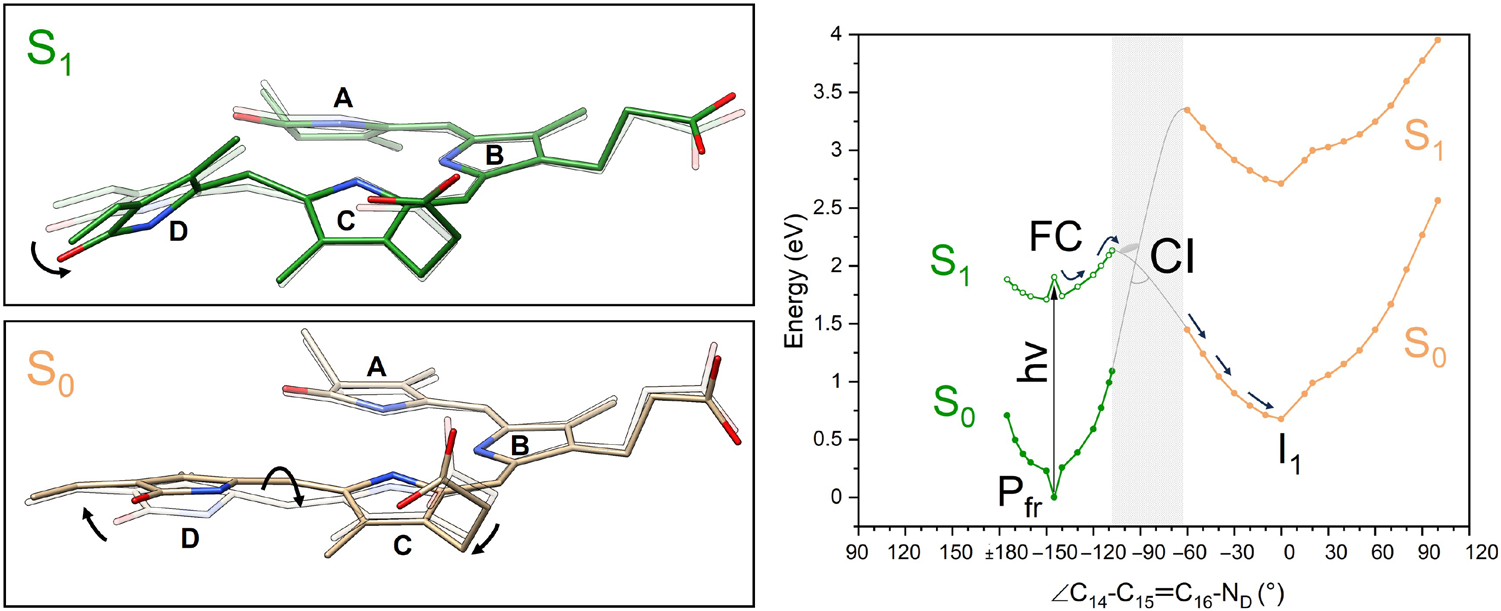
QM/MM relaxed scan of the Agp2-PAiRFP2. (**Left**) Top: The chromophore structures corresponding to the FC geometry (∠ = −150°, light green) and the final geometry (∠ = −108°, dark green) of the S1 relaxed scan. Bottom: The chromophore structures along the S0 relaxed scan corresponding to the ground state geometry after the CI region (∠ = −60°, gray) and the minimum of the S0 of the early event in I1 (∠ = 0.0°, tan). (**Right**) Evolution of the photoreaction from the Pfr state (I0a) to I1 where the initial excitation is from the QM/MM optimized Pfr S0 minimum to the FC geometry. The potential energy curves which correspond to initial coordinates obtained from the Pfr I0a structure are shown in green, and those from the I1 structure are shown in yellow. The open and closed circles of the S0 and S1 Pfr curves correspond to S0 energies obtained from the S1 optimizations and the optimized S1 energies, respectively. The S0 I1 curve corresponds to optimized S0 geometries, and the S1 curve corresponds to their vertical excitation energies.

The availability of the Agp2-PAiRFP2 atomic model in the resting state to 2.15-Å resolution and a 2.54-Å resolution SFX atomic model for the I1 dataset allows us to investigate the photoisomerization pathway using hybrid quantum mechanics/molecular mechanics (QM/MM) simulations (Fig. 4). We have performed a relaxed scan in the excited state starting from the resting state (Fig. 4, left) by twisting the C_14_-C_15_═C_16_-N_D_ dihedral angle. A rotation of ring *D* in the clockwise direction leads to a decrease in the S_1_-S_0_ energy gap and is compensated by the puckering of the methine bridge in the opposite direction. The rotation in the counterclockwise direction leads to a clash between the methyl groups of the rings *C* and *D*, associated with a high increase in energy. The ground state pathway that leads to the major structural component modeled in the I1 dataset (Fig. 4, right) is characterized by a rotation from 60° to 0° of the C_14_-C_15_═C_16_-N_D_ dihedral angle. In this step, there is a large rotation of ring *C* which is moving in the opposite sense of rotation with respect to ring *D*. Because of the movement of ring *C*, there is also a large displacement of the propionate at the same ring.

### Group 2 of molecular events (30 to 100 ms)—Structural relaxation of the chromophore and adaptations of the hydrogen bond network

**Fig. 5.**
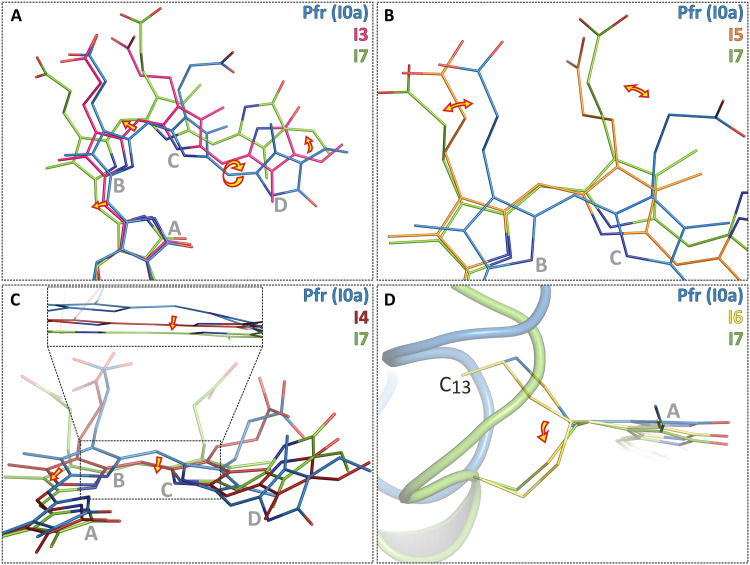
Close-up view of the stepwise structural changes of BV during the Pfr photoconversion of Agp2-PAiRFP2. (**A**) Photoisomerization of the chromophore, followed by thermal relaxations including the three methine bridges *C*-*D*, *B-C*, and *A-B*. (**B**) Relaxation of the propionic side chains of rings *B* and *C*. (**C**) Movement of the BV skeleton due to an increasing coplanarity of the rings *B* and *C* corresponding to a decreasing torsion (tilt) angle of the *B*-*C* methine bridge (Fig. 3). (**D**) Facial change of the chromophore attachment to Cys^13^. BV and Cys^13^ are shown as lines and the protein backbone as a cartoon. The Pfr structure (I0a) is colored blue, the I7 structure is colored green, and selected intermediate structures are colored pink (I3), red (I4), orange (I5), and yellow (I6).

The relaxation of the isomerized chromophore involves the *prop-B* and *prop-C* as verified by the polder-omit (*36*) and isomorphous *F*o-*F*o electron density maps (*37*). Already in I1 two main conformations of *prop-B* were observed (Fig. 2 and fig. S2). One conformation is only slightly different from the Pfr state but with a similar hydrogen bonded interaction to Arg^211^ and water-mediated to Tyr^205^ and Arg^242^. However, the surrounding water molecules show increased mobility (fig. S7). The second conformation is shifted more toward Arg^242^. In I2, *prop-B* completely reverts to the Pfr structure concomitant to the photochemical back isomerization (Fig. 2 and figs. S1, S2, and S7), and the corresponding hydrogen bonding partners do not display any structural changes. Subsequently, a shift of *prop-B* is again observed, and lastly, it undergoes a bidentate binding with Arg^242^ as the only conformation in I5, similar to the SR structure of the Meta-F state previously observed (fig. S8) (*19*). In this sense, *prop-C* shows a similar behavior. In Pfr, it is located in hydrogen bond distance to Tyr^165^, His^278^, a conserved water, and in one dataset (I0b) additionally to His^248^. Starting with I3, 15% of *prop-C* shifts into an altered but transient conformation, in which it forces first structural changes in the protein environment. Here, Tyr^205^ is displaced as the first step of the amino acid rearrangement in the CBP. This conformation of Tyr^205^ in turn allows Arg^211^ to rotate into its position of the late Meta-F state (I7) (Fig. 2 and figs. S8 and S9). These structural adaptations are accompanied by the rearrangement of water molecules, which is completed in I6. However, the final conformation of *prop-C* appears for the first time in I5, whereas the final hydrogen bond network is established in I6 (Fig. 2 and figs. S1 and S2). These conformational changes which are most likely a consequence of the rearrangement of the hydrogen bond network due to the isomerization (Fig. 5, A and B) are accompanied by geometrical changes of the BV skeleton. Aligning ring *A* in Pfr and the intermediate states, we note shifts of the other pyrrole rings that are largest for ring *D* but extend even to ring *B*. These structural changes toward an increased coplanarity of the four pyrrole rings (Fig. 5C) continue up to the final photoproduct (I7) and can be expressed by the torsional (tilt) angles of the three methine bridges that fluctuate and then decrease (for the torsional angles *B*-*C* and *A*-*B*) from I1 to I7 (Fig. 3).

### Group 3 of molecular events (140 to 170 ms)—Rearrangements in the CBP

The structural rearrangement of the amino acids in the CBP starts in I3 (100-ms irradiation and 50-ms delay) and becomes the dominant event in the subsequent time frames. The positions of Asp^196^, His^248^, and the pyrrole water, which are close to the pyrrole N-H groups of rings *A*, *B*, and *C*, are adjusted as a consequence of the *C*-*D* methine bridge rotation. These adjustments are accompanied by slight displacements of helices 8 and 9 (figs. S11 and S12). The refined position of the pyrrole water changes and becomes more disordered up to I5 as reflected in the reduced *m*Fo-*D*Fc peak height (table S7). In prototypical phytochromes, this water molecule was found to be transiently removed from the pyrrole in the excited state (*31*), but such a movement does not seem to take place here. In I7 (6-s irradiation and 360-s delay), the electron density maps indicate a restabilization of this water.

In addition to the events in group 2, further strong signals appear in group 3 in the electron density map, namely, the displacements of the aromatic amino acids Tyr^165^ and Phe^192^ adjacent to ring *D* (Fig. 2 and figs. S13 and S14). BV isomerization causes the ring *D* carbonyl to interfere with the original Pfr hydrogen bonding network involving Tyr^165^ and His^278^ (fig. S15). This interference destabilizes the interactions of *prop-C* with Tyr^165^, which eventually leaves this network as reflected by the steady decrease in electron density. Instead, Tyr^165^ rotates to the original position of Phe^192^ which in turn is shifted to an altered position, supported by hydrophobic interactions with ring *D*. The increasing coplanarity of BV rings *C* and *D* seems to assist this stabilization of Tyr^165^ (Figs. 2 and 5, A and C), a process that is further supported by an additional water molecule at very late times (I7; fig. S16).

### Group 4 of molecular events (3 to 6 s)—Structural changes of the protein backbone

I6 (2.5-s irradiation and 360-s delay) is the first dataset in which conformational changes of the protein backbone are observable, involving the N-terminal part and the tongue. The BV binding site Cys^13^ displays a double conformation with an α- and β-facial orientation (Fig. 5D and figs. S17 and S18), and the N terminus partially refolds. The transition to the final β-facial orientation is completed in I7 (Figs. 2 and 3, figs. S17 and S18, and tables S3 and S4).

In the time frame I6, we observe the onset of the refolding of the tongue (fig. S19). It is initiated by the outward displacement of Gln^190^, starting already in group 3, together with the tongue residue Trp^440^, which has a reduced electron density and lastly leaves the original hydrogen bond network in I7 (fig. S20). Furthermore, two water molecules, hydrogen bonded to Gln^190^ in Pfr, are destabilized during this movement, but one of them is restabilized in an altered position during the transition from I6 to I7. As a result, the hydrogen bond network of Gln^190^ is altered, and Trp^440^ reorients, further increasing the distance to Gln^190^. In contrast to our previous results for the trapped Meta-F state of Agp2-PAiRFP2 (*19*), the unresolved portion of the tongue is rather small in I6 and includes the region 441 to 445, which, in Pfr, adopts a loop structure at the beginning of the tongue. In I7, a refolding of this segment is observed such that the only unresolved residue is 442 (figs. S12, S19, and S20). Note that the experimental conditions for I6 and I7 are quite similar. It may be that the differences between these time frames just reflect the structural fluctuations in the final Meta-F state, consistent with the RR spectroscopic results of the cryo-trapped Meta-F state (*19*).

## DISCUSSION

### Conversion of light energy into structural changes

**Fig. 6.**
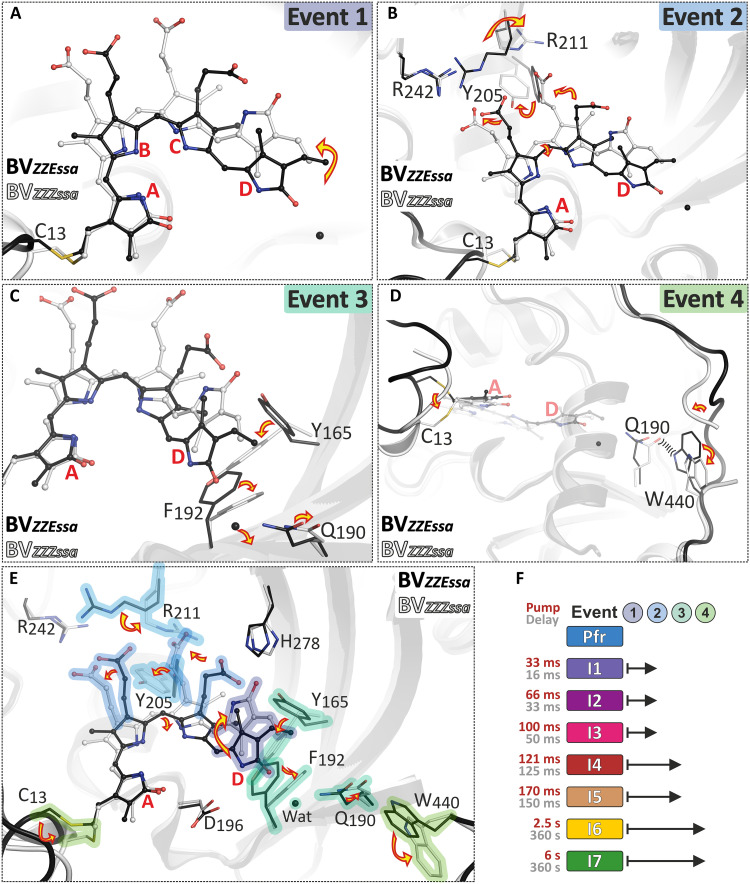
Sequence of events during the Pfr photoconversion of Agp2-PAiRFP2. All figures show a superposition of the structures of Pfr I0a (black) and the final state I7 (white). (**A**) Event 1 is the isomerization of the chromophore from the *ZZEssa* to the *ZZZssa* configuration. (**B**) Event 2 is the relaxation of the BV skeleton and the *prop-B* and *prop-C* side chains. This is coupled to structural changes at Tyr^205^ and Arg^211^ and accompanied by a reduction of the torsional angle of the *C-D* methine bridge. (**C**) As a result, in event 3, Tyr^165^ and Phe^192^ rotate, a water molecule is released from the CBP, and Gln^190^ moves together with Trp^440^ from its original position. Structural changes (in form of a restructuring) of the protein backbone occur in the late part of the photoconversion and represent event 4 (**D**). Structural changes at the N terminus and the tongue region (event 4) occur on a similar timescale. (**E** and **F**) Overview of the structural rearrangements at the chromophore and its binding pocket during the photoconversion within different illumination and relaxation times. The chromophore is depicted as balls/sticks, and the protein backbone is depicted as cartoon and specific amino acids as sticks. The water molecule is shown as black sphere.

Photoisomerization of BV (event 1; Fig. 6) affects the immediate contacts, first at ring *D* and subsequently on the rest of the chromophore, on a very short timescale beyond the resolution of the present experiments (*23*). The subsequent thermal relaxations that start at the chromophore include fluctuations and lead to a decrease of methine bridge torsions in *C-D* and *B-C* as well as subsequently in *A-B* (Fig. 3). This process is accompanied by the reorientation of the *prop*-*B* and *prop*-*C* side chains as well as water molecules in the CBP (event 2). With the final steps of *prop*-*B* and *prop*-*C* toward their equilibrium positions in I6 and the progressive chromophore relaxation, additional space in the CBP is created for structural and orientational changes of the amino acids in the vicinity of rings *B*, *C*, and *D* starting in I3 (Tyr^205^ and Arg^211^ for event 2), I4 (Tyr^165^, Phe^192^, and His^278^ for event 3), and I5 (Gln^190^ for event 3).

### Proton transfer and tongue structure transformation

The relaxation of the chromophore and the CBP is a mutual displacement process, which is the steric prerequisite for the proton transfer from *prop-C* to His^278^ occurring in Agp2-WT with the decay of Meta-F to Pr (*16*). The additional requirement for proton transfer is the lowering of the p*K*_a_ (where *K*_a_ is the acid dissociation constant) of the protonated *prop-C* from approximately 11 in Pfr to a value lower than 7. This can be achieved by changes in local electrostatics following chromophore isomerization. In Agp2-PAiRFP2, the p*K*_a_ remains too high to allow for a proton transfer as in Agp2-WT which is essential for the secondary structure transformation of the tongue (*20*). It is initiated by a conformational switch (event 4) involving the displacement of Gln^190^ and Trp^440^ (I7), which undergoes a reorientation, thereby restructuring the loop portion of the tongue. The rearrangement of the N terminus (in Mol A) seems to be synchronized with this conformational switch (event 4) and the preceding structural changes in the CBP (event 3) since a restricted mobility [due to packing of the N-terminus (Mol B)] causes a slower and incomplete photoconversion. This finding is consistent with previous results reporting a cross-talk between the N-terminal segment and the PHY tongue region (*38*). Last, we like to point out that the coupling mechanism between chromophore photoisomerization, proton transfer, and protein (tongue) structural changes is not simply a reversal of the photoinduced conversion from Pr-to-Pfr since, in that case, proton transfer involves the pyrrole N-H groups of BV (*12*). However, in both photoconversion routes of all phytochromes, chromophore isomerization is coupled to protein structural changes via intramolecular proton transfer.

In summary, the present series of structural snapshots collected after different illumination and incubation times allow for a detailed description of the sequence of structural changes following the photoisomerization of a phytochrome (Fig. 6). It is shown that the structural changes proceed synchronously with increasing time and length scales, starting with the relaxation of the chromophore and its immediate environment at early times, followed by major adjustments of amino acids in the CBP which in turn induce the conformational switch to initiate tongue restructuring. The results provide insight into the coupling of the chromophore with the protein structural changes that constitute a crucial step in the (de)activation process of the photoreceptor. Thus, both the results and the conceptual and methodological approach are important in the broader context of understanding the molecular functioning of phytochromes and studying photoreceptors in general.

## MATERIALS AND METHODS

### Molecular cloning of Agp2-PAiRFP2

The amino acid sequence of Agp2-PAiRFP2 (NCBI GenBank ID AGS83373.1) (*19*, *28*), a derivate of Agp2 from *A. fabrum*, was codon optimized for expression in *Escherichia coli* (*39*). The gene was synthesized by GENEWIZ Inc. and cloned into pET21b expression vector with C-terminal His-tag and transformed into *E. coli* BL21-DE3. The construct contains the following 24 substitutions: Lys^69^Arg, Arg^83^Lys, Gly^120^Asp, Ala^123^Thr, Met^163^Leu, Gln^168^Glu, Arg^220^Pro, Ser^243^Asn, Val^244^Phe, Gly^269^Asp, Ala^276^Val, Tyr^280^Cys, Glu^294^Ala, His^303^Phe, His^333^Arg, Ile^336^Leu, Asp^349^Arg, Met^35^1Ile, Ala^386^Val, Gly^409^Asp, Leu^419^Ile, Thr^469^Ser, Ala^487^Thr, and Glu^494^Gly.

### Purification of Agp2-PAiRFP2

Agp2-PAiRFP2 constructs were expressed using an autoinduction medium (Overnight Express Instant TB Medium; Novagen) for 48 hours and 20°C. Cell pellets were washed, and cell lysis was carried out using a cell fluidizer (Microfluidics, Newton, USA) in 50 mM tris-HCl buffer containing 50 mM sodium chloride at pH 7.8, 5% glycerol, lysozyme (2 mg/ml; Merck Millipore), deoxyribonuclease (60 μg/ml; Sigma-Aldrich), 1 mM MgCl_2_, and 0.5 mM phenylmethanesulfonyl (Sigma-Aldrich). Lysed cells were centrifuged, and the supernatant was precipitated with 2 M ammonium sulfate. The cell pellet was eluted with 50 mM tris/HCl, 10 mM imidazole, and 400 mM sodium chloride at pH 7.8 and loaded on a nickel-nitrilotriacetic Acid (Ni-NTA) column [5-ml high performance (HP) columns; GE Healthcare]. Purified apo-phytochrome was eluted with a linear imidazole gradient. Imidazole was removed by ammonium sulfate precipitation. The chromophore BV (Frontier Scientific) was added to the protein in approximately three molar excess. The holoprotein was precipitated with ammonium sulfate and resuspended in size exclusion buffer [20 mM Hepes buffer (pH 7.5) and 150 mM sodium chloride]. Size exclusion chromatography (HiLoad Superdex 200 column, GE Healthcare) yielded pure protein with a concentration of 30 mg/ml.

### Crystallization of Agp2-PAiRFP2

Agp2-PAiRFP2 was crystallized in the dark at 6°C in a precipitation solution containing 1.0 to 2.2 M ammonium sulfate, 2 to 12% polyethylene glycol, molecular weight 1000, 0.1 M Hepes (pH 6.8 to 7.7), and 0.025% low-melt agarose. First crystals appeared after 1 day reaching an average size of 50 μm by 50 μm by 20 μm after 1 to 2 weeks.

### Sample delivery and illumination experiments of Agp2-PAiRFP2

The drop-on-tape (DOT) sample delivery system (*40*) was used in combination with the acoustic droplet ejection (ADE) to collect structural data on Agp2-PAiRFP2. To prevent degradation of crystal quality, the samples were kept at 4°C in the Hamilton syringe used for sample delivery, and the capillary connecting the delivery syringe to the ADE droplet generator was cooled to 12°C by a water sleeve, which also provided protection against stray light. In addition, the ADE droplet generator was extended to include a cooling sleeve that was kept at 12°C, allowing the sample to be kept below room temperature until the moment when the droplet of crystal slurry was generated.

For photoconversion, experiments and capturing intermediate states Agp2-PAiRFP2 crystals were irradiated at either of three illumination points (see table S1 for the summary of illumination conditions). Illumination point 1 (ILP1) was located on the Kapton belt 5 mm upstream of the x-ray interaction point and had a spot size of 10 mm on the tape, allowing the samples to be illuminated on the short tens of millisecond timescale. ILP2 was located 15 mm upstream of the x-ray interaction point and had a spot size of 17 mm on the Kapton belt, allowing for illumination times and for time delays between illumination and x-ray probe in the range of 100 ms. ILP3 was located in the sample delivery capillary before the droplet generator, allowing delays between illumination and the x-ray probe of several minutes. Continuous light-emitting diode (LED) light sources were used at all three points, and a wide range of illumination times can be achieved by adjusting the size of the light spot on the Kapton belt or capillary together with the belt speed or sample flow rate through the capillary.

For photoconversion, experiments and capturing intermediate states Agp2-PAiRFP2 crystals were irradiated either with a 785-nm LED (Thorlabs Inc., New Jersey, USA) at LCLS (Stanford, USA) or with a 770-nm LED (pE4000, CoolLED, UK) at SACLA (Kouto, Japan). Illumination at points 1 and 2 was at 25°C, while for illumination at position 3, the cooling sleeve had a gap just outside of the DOT setup where the capillary rested on a cooling block and was covered with a variable size aperture allowing illumination at 12°C. Illumination times could be varied by adjusting the length of the capillary to be illuminated (via a circular aperture with different diameters) and by changing of the flow rate (table S1).

### Data collection and structure analysis

Data collection was performed at room temperature (typically ~25° to 27°C inside the hutch) at the MFX-beamline at LCLS (Stanford, USA) (*41*–*43*) during the LS00 and LS34 experiments at λ = 1.301 Å. Agp2-PAiRFP2 crystals were measured using x-ray pulses of 40-fs length with an x-ray spot size at the sample of 3 to 4 μm in diameter (full width at half maximum) using compound refractive lenses and a pulse energy of 2 to 3 mJ. The atmosphere surrounding the tape drive sample delivery apparatus was approximately 100% He. X-ray diffraction data were collected on a Rayonix MX170 HS detector operating in 2-by-2 binning mode at a maximum frame rate of 10 Hz. The SACLA data collection (proposal 2018B8076) at the BL2/EH3 hutch (*44*, *45*) was conducted at λ = 1.24 Å at room temperature (typically ~29° to 32°C inside the hutch) using 10-keV x-ray photons, a pulse length of ~10 fs, approximately 400 μJ per pulse, delivered at 30 Hz, and focused to ~3 μm at the sample. The atmosphere surrounding the tape drive sample delivery apparatus was a mixture of 50% He in air, and a light tube filled with 100% He was used upstream of the x-ray interaction point and a beam stop downstream of the interaction point to minimize background scattering on the detector. SACLA diffraction data were recorded on the octal multiport readout charge-coupled device (MPCCD) detector with online data evaluation via cctbx.xfel working from the hits provided by the CHEETAH pipeline at the beamline (*46*, *47*).

The cctbx.xfel graphical user interface was used to track the acquisition of the data collection, providing real-time feedback and submitting processing jobs (*48*). Data processing was performed using the program dials.stills_process to perform lattice indexing, crystal model refinement, and integration (*49*–*55*). First, strong spots were selected for unit cell parameter calculations. A crystal model (consisting of a unit cell and crystal orientation) was then refined to minimize differences between observed spot centroids and predicted positions. This model was used to generate a complete set of predicted reflection positions on the frame. Last, signal at these positions was integrated, and any corrections or uncertainties were taken into account. A powder diffraction pattern of a silver(I) behenate sample (Alfa Aesar) pressed between two sheets of Kapton tape was used to make an initial estimate of the detector distance. Initial indexing results were used to refine the detector distance and position for each interval between adjustments to the sample delivery system or detector position. These more accurate detector positions were used in the indexing and integration trails, resulting in a maximum of four distinct lattices indexed on a single shot. Cluster.unit_cell, a command line tool in cctbx that clusters similar unit cells according to the Andrews-Bernstein distance metric (*56*–*58*), was used to obtain the average unit cell. This unit cell was taken as the target unit cell when reprocessing all experimental data with dials.stills_process.

A total of 251,018 integrated crystal lattices from the different datasets were obtained using dials.stills_process with a target unit cell of *a* = 182.4 Å, *b* = 182.4 Å, *c* = 179.9 Å, α = 90°, β = 90°, γ = 120° and hexagonal space group P6_3_22 [for I0a (LCLS) 45,331 lattices; I0b (SACLA) 14,431; I1 (LCLS) 12,794; I2 (LCLS) 23,308; I3 (LCLS) 49,031; I4 (SACLA) 23,055; I5 (SACLA) 22,367; I6 (LCLS) 22,216; I7 (LCLS) 38,485; total lattices merged overall 251,018].

Diffraction spots were integrated to the edges of the detector in anticipation of a resolution cutoff per image during the merging step. The integrated intensities were corrected for absorption by the Kapton conveyor belt to match the position of the belt and crystals relative to the x-ray beam (*40*).

The integrated crystal lattices were merged using cxi.merge as previously described (*59*), with a few modifications. The default unit cell outlier rejection mechanism in cxi.merge was sufficiently selective on the image set curated as described above, so a prefiltering step was not required. In addition, a reference model and dataset with a compatible unit cell—used by cxi.merge during scaling—were available from previous beam times. Merged datasets were acquired for the dark-adapted Pfr state, after 30-, 60-, and 100-ms illumination at ILP1 (LCLS, Stanford, USA). Furthermore, data were collected and merged for a dark-adapted Pfr state, 140- and 170-ms illuminated dataset at illumination point 2 (SACLA, Kouto, Japan). In addition, merged datasets were collected after 3- and 6-s irradiation at illumination point 3. Datasets contained between 12,794 and 49,031 images (tables S2, S5, and S6) with a resolution between 2.15 and 2.80 Å. Initial phases for Agp2-PAiRFP2 were obtained by molecular replacement with Phaser (*60*) (rotation, translation, and rigid-body fitting) using the Agp2-PAiRFP2 in its Pfr state [Protein Data Bank (PDB) ID 6G1Z] as the initial search model (*19*) (excluding BV from initial search models). A simulated annealing procedure was performed on the resulting model using a slow-cooling protocol and a maximum likelihood target function, energy minimization, and B-factor refinement by the program Phenix (*37*). After the first round of refinements for all datasets, the BV in the ligand binding pocket was visible in the electron density of both σ_A_-weighted 2*m*Fo-*D*Fc maps, as well as in the σ_A_-weighted simulated annealing omitted density maps for BV chromophores (figs. S1 and S2). The I6/Meta-F dataset was modeled using TLS (Translation-Libration-Screw-rotation) refinement (*61*) with anisotropic temperature factors for all protein atoms. For all crystal structures restrained, individual *B*-factors were refined, and the crystal structure was finalized using the CCP4 program REFMAC5 (*62*) and other programs of the CCP4 suite (*63*). Final agreement factors *R*_free_ between 21.7 and 32.1% and *R*_cryst_ between 20.2 and 27.6% were obtained (tables S2, S5, and S6). Manual rebuilding of the crystal structure models and electron density interpretation were performed after each refinement cycle using the program COOT (*64*). Structures were validated with the programs Phenix (*37*), SFCHECK (*65*), WHAT_CHECK (*66*), MolProbity (*67*), and RAMPAGE (*68*). Potential hydrogen bonds and van der Waals contacts were analyzed using the programs HBPLUS (*69*) and LIGPLOT 1.45+ (*70*). All ΔFo electron density maps were calculated with Phenix (*37*). All other electron density maps were calculated with the CCP4 program FFT (*71*). All crystal structure superpositions of backbone α-carbon traces were performed using the CCP4 program LSQKAB (*63*). The final resolution of all “states” (PDB entries in parentheses) are as follows: I0a (8RJM), 2.15 Å; I0b (8RJN), 2.20 Å; I1 (8RJO), 2.54 Å; I2 (8RJP), 2.43 Å; I3 (8RJQ), 2.40 Å; I4 (8RJR), 2.30 Å; I5 (8RJS), 2.43 Å; I6 (8RJT), 2.49 Å; I7 (8RJU), 2.80 Å. All molecular graphic representations were created with PyMOL (*72*).

### Computational details

#### Model preparation

The computational model of the resting state (Pfr) was generated from chain A of the Agp2 crystal structure (PDB ID 8RJM) reported as I0a dataset in this work. The missing amino acid residues between SER121-GLN124 and LEU78-THR84 were added via the Modeller extension (*73*, *74*) implemented in UCSF Chimera (*75*). The I_1_ model for the photoproduct was generated from chain A of the 33-ms illuminated structure from this work (PDB ID 8RJO). There were no missing residues in this model. Hydrogens were added to the models using the tleap program considered a pH of 7.0 for the protonation state of the titratable residues. The histidine residues near the BV chromophore, His^248^ and His^278^, were protonated at the epsilon position. The crystal waters were retained for all simulations. After protonation, the models underwent energy minimization with AMBER16 (*76*).

#### QM/MM geometry optimizations

The Pfr (I0) and I1 models were optimized using the hybrid QM/MM method (*77*). The optimization was carried out at the density functional theory (DFT) level of theory with the B3LYP (*78*, *79*) functional and the cc-pVDZ (*80*) basis set with dispersion corrections (*81*) and Becke-Johnson damping (*82*–*84*) included. The MM region was treated with the AMBER ff14sb force field (*85*). The QM region contained a truncated BV chromophore of 60 atoms with 3 link atoms totaling 63 atoms. The chromophore was truncated at the C─C bond (named C_AC_ and C_BC_ in the crystal structure) of the ring A side chain at the connection to the cysteine. Another QM-MM boundary was placed at the propionate side chains of ring *B* and *C* between the sp2 carbon of the ring and the sp3 carbon of the side chain. A QM region of this size has been shown to be suitable for excited state calculations involving phytochromes because it does not truncate the conjugated ring system (*86*). Any residue or water molecule which had an atom within 5 Å of the chromophore was allowed to freely move throughout the optimization. The QM/MM optimizations were carried out with ChemShell (*87*, *88*) interfaced with Orca, and the L-BFGS (*89*) approximation was used in the internal DL-FIND (*90*) module of ChemShell while the DL-POLY module was used for the MM portion.

#### S_1_ excited state relaxed scan

The QM/MM optimized models were used as the starting point for the S_1_ excited state surface scan along the isomerization of the double bond between rings *C* and *D*. The ChemShell DL-FIND module was used for the optimization, and the adiabatic excitation energies were computed with the algebraic diagrammatic construction to the second order [ADC(2)] (*91*, *92*) with the cc-pVDZ basis set in Turbomole 7.0 (*93*, *94*). All other details of the relaxed scan QM/MM simulations were kept consistent with the geometry optimizations described above. The dihedral angle between ring *C* and *D* (∠C_14_-C_15_═C_16_-N_RingD_) was systematically increased by 10° in both the clockwise and counterclockwise directions until the limit of the single-reference ADC(2) method was reached. ChemShell interfaced with Turbomole 7.0 was used for the excited state calculations.
