# Supplementary material for: Serial-femtosecond crystallography reveals how a phytochrome variant couples chromophore and protein structural changes
Source: Sci Adv. 2025 May 28;11(22):eadp2665. doi: 10.1126/sciadv.adp2665 (PMC12961760; doi:10.1126/sciadv.adp2665)
Supplement: Supplementary file 1 — Figs. S1 to S22 Tables S1 to S7 Legend for movie S1 [file sciadv.adp2665_sm.pdf]

Science Advances
AAAS

Supplementary Materials for

Serial-femtosecond crystallography reveals how a phytochrome variant couples chromophore and protein structural changes

Luisa Sauthof *et al.*

Corresponding author: Patrick Scheerer, patrick.scheerer@charite.de

Sci. Adv. **11**, eadp2665 (2025)
DOI: 10.1126/sciadv.adp2665

The PDF file includes:

Figs. S1 to S22
Tables S1 to S7
Legend for movie S1

Other Supplementary Material for this manuscript includes the following:

Movie S1

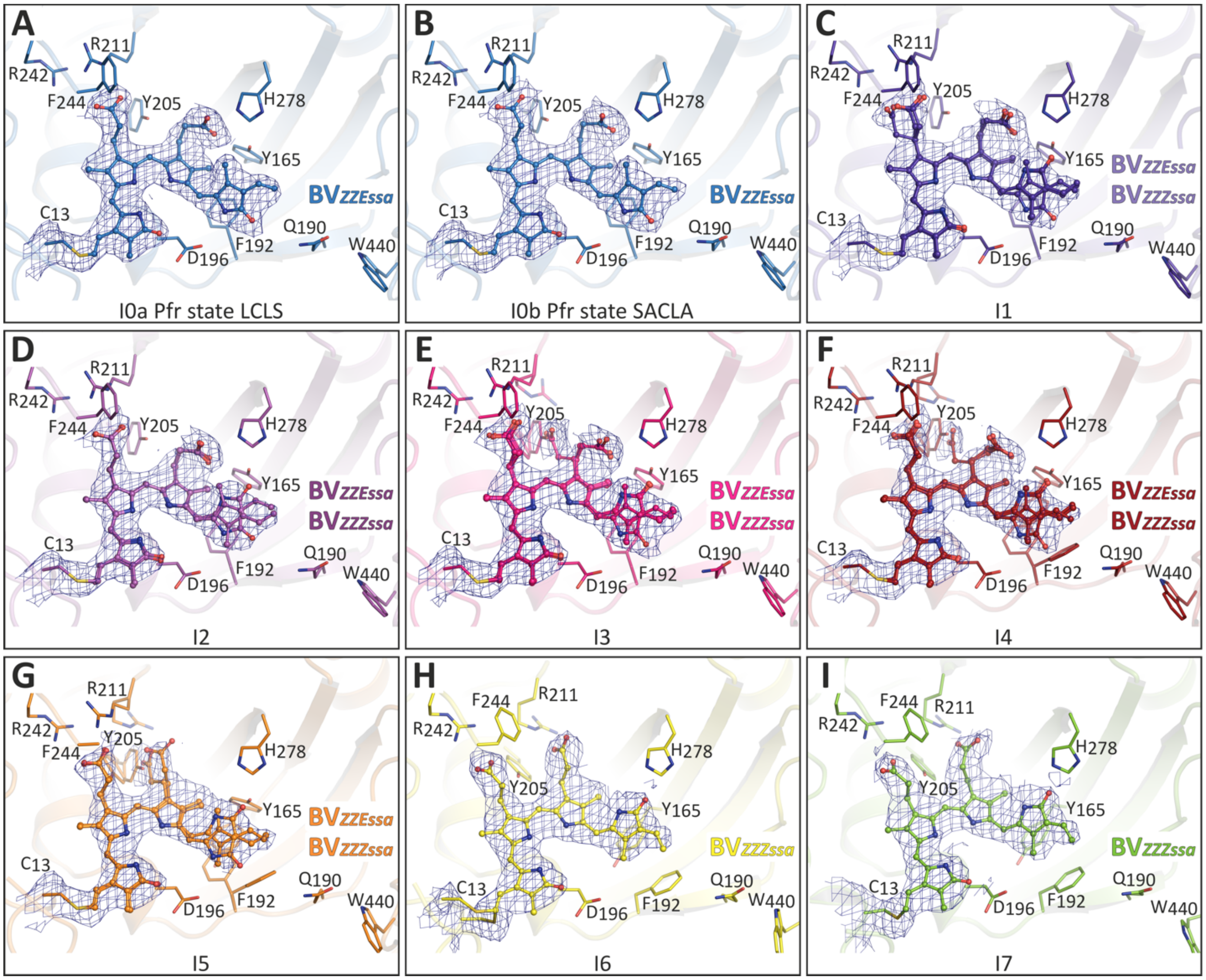

Fig. S1. Quality of the 2*m*Fo-*D*Fc electron density of the chromophore of Agp2-PAiRFP2. Close-up view of the chromophore and its binding pocket of Agp2-PAiRFP2 in the times frames as defined in Table S1. (**A-B**), **I0a** and **I0b** (Pfr state) with biliverdin (BV) and protein colored in blue; data collected at LCLS PDB-ID 8RJM and SACLA PDB-ID 8RJN, respectively; (**C**), **I1** (violet, PDB-ID 8RJO); (**D**), **I2** (purple, PDB-ID 8RJP), (**E**) **I3** (pink, PDB-ID 8RJQ), (**F**) **I4** (dark-red, PDB-ID 8RJR), (**G**) **I5** (orange, PDB-ID 8RJS), (**H**) **I6** (yellow, PDB-ID 8RJT), (**I**) **I7** (green, PDB-ID 8RJU). All figures show the 2*m*Fo-*D*Fc electron densities of the chromophore (dark-blue meshes) contoured at 1.0 σ level. BV, the protein backbone and selected amino acids are depicted in balls and sticks, cartoon and stick representation, respectively.

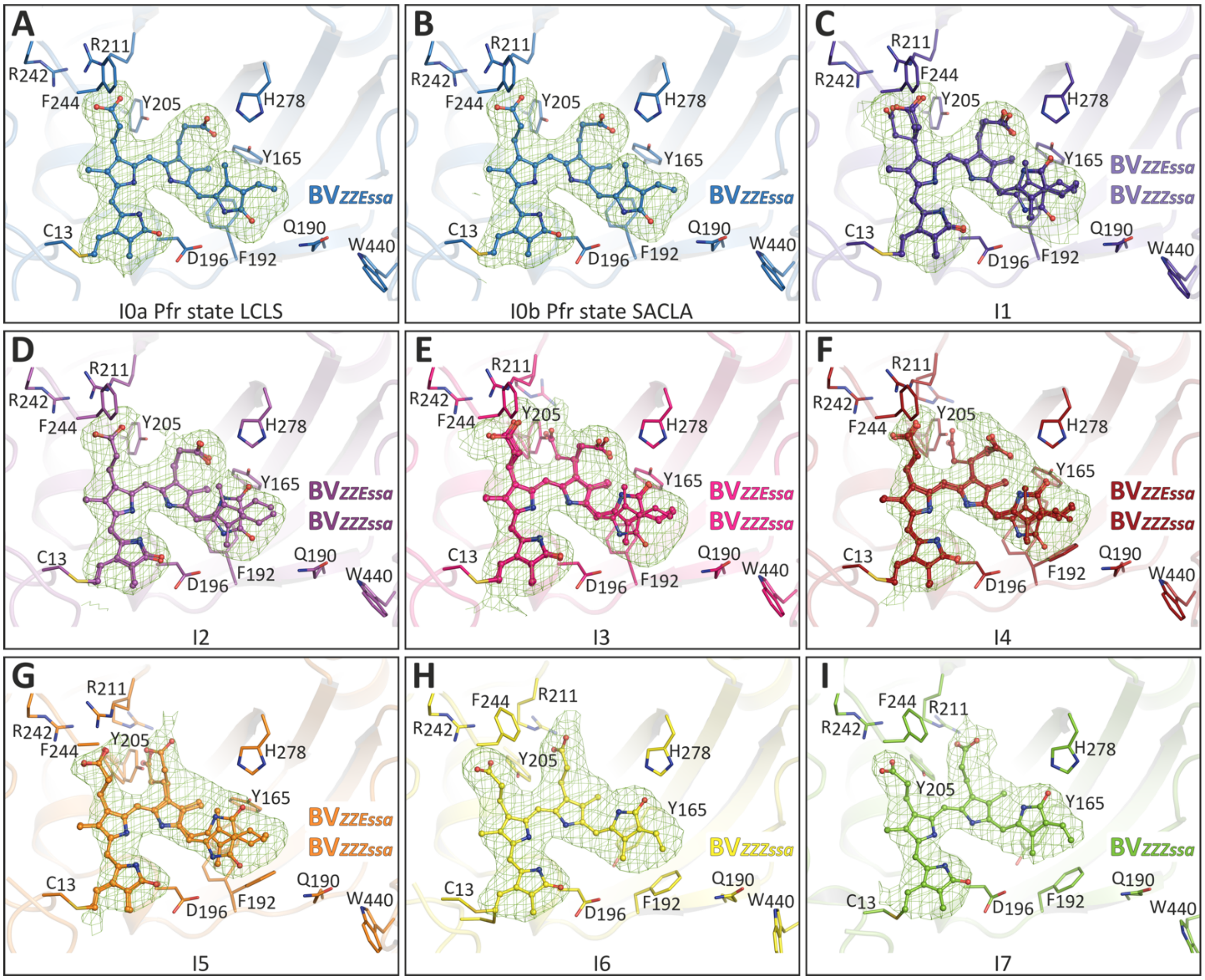

Fig. S2. Polder omit maps of the chromophore of Agp2-PAiRFP2 at different time frames in the light-activation process.

Close-up view of the chromophore and its binding pocket of Agp2-PAiRFP2 in the time frames as defined in Table S1. (**A**-**B**), **I0a** and **I0b** (Pfr state) with biliverdin (BV) and protein colored in blue; data collected at LCLS and SACLA, respectively; (**C**), **I1** (violet); (**D**), **I2** (purple), (**E**) **I3** (pink), (**F**) **I4** (dark-red), (**G**) **I5** (orange), (**H**) **I6** (yellow), (**I**) **I7** (green). The "polder" *m*Fo-*D*Fc omit electron density maps for BV are contoured at 3.0 σ (green meshes) and calculated with the program phenix.polder (*35, 36*). BV was omitted in the calculation as well as bulk solvent around

the ligand region. This procedure often yields improved electron density in the region of the ligand region that is not obscured by bulk solvent anymore. BV, the protein backbone and selected amino acids are depicted in balls and sticks, cartoon and stick representation, respectively.

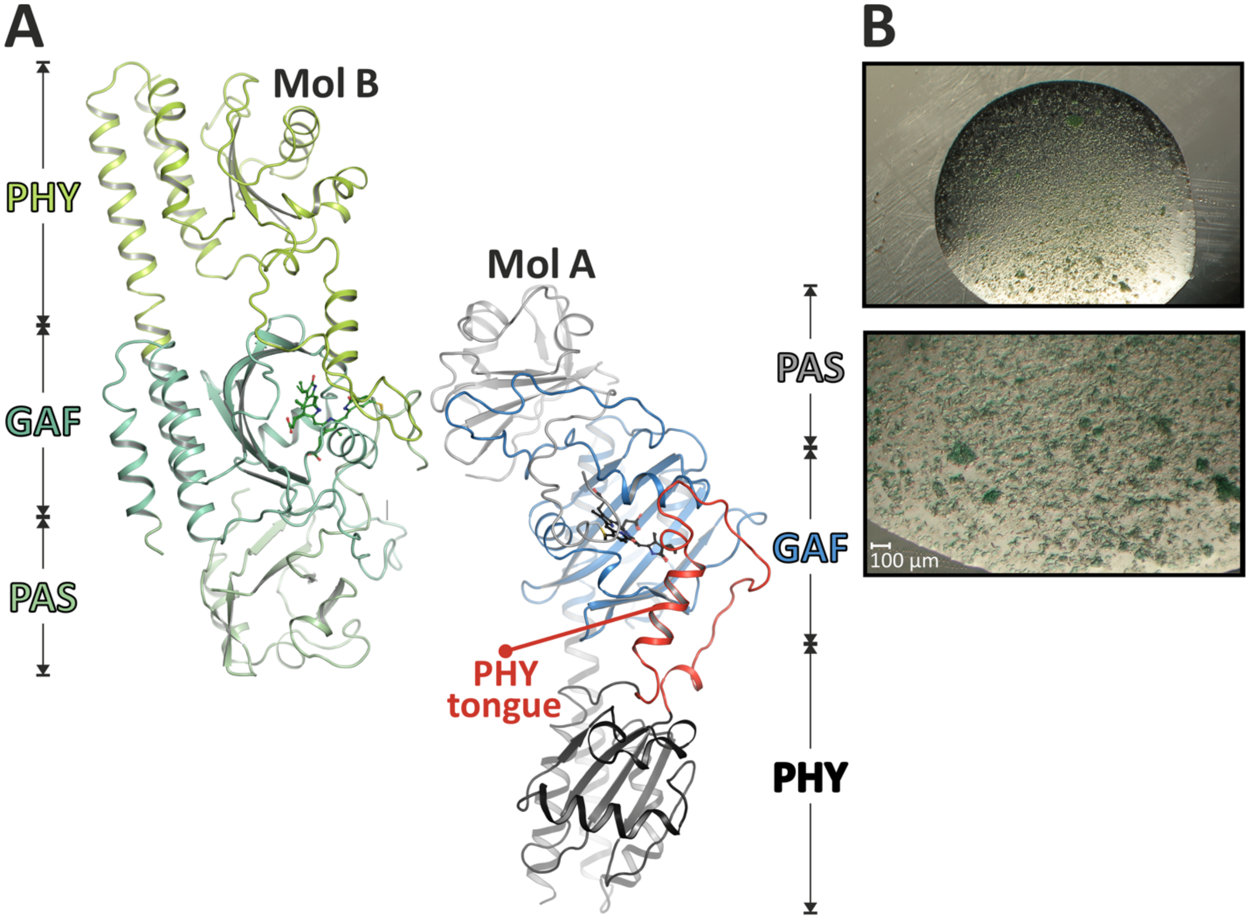

Fig. S3. Crystallographic dimer packing and representative microcrystals of Agp2-PAiRFP2.

(**A**) Crystallographic symmetry monomers (Mol A and Mol B) in the asymmetric unit for Agp2-PAiRFP2. The Agp2-PAiRFP2 construct consists of three different domains (PAS (Per/Arndt/Sim-), Mol A grey; GAF (cGMP phosphodiesterase/adenyl cyclase/FhlA-), Mol A blue; PHY (phytochrome-specific-), Mol A black) comprising the highly conserved photo-sensory core module (PCM). Agp2-PAiRFP2 crystallizes in the hexagonal space group $P6_322$ with a nearly antiparallel monomer packing. BV and the protein backbone are depicted in balls and sticks and cartoon representation, respectively. Mol B is colored in shades of green. (**B**) shows a

representative drop of micro crystals measured at LCLS (Stanford, USA) and SACLA (Kouto, Japan). In average the crystal size was 50 x 50 x 20 µm³.

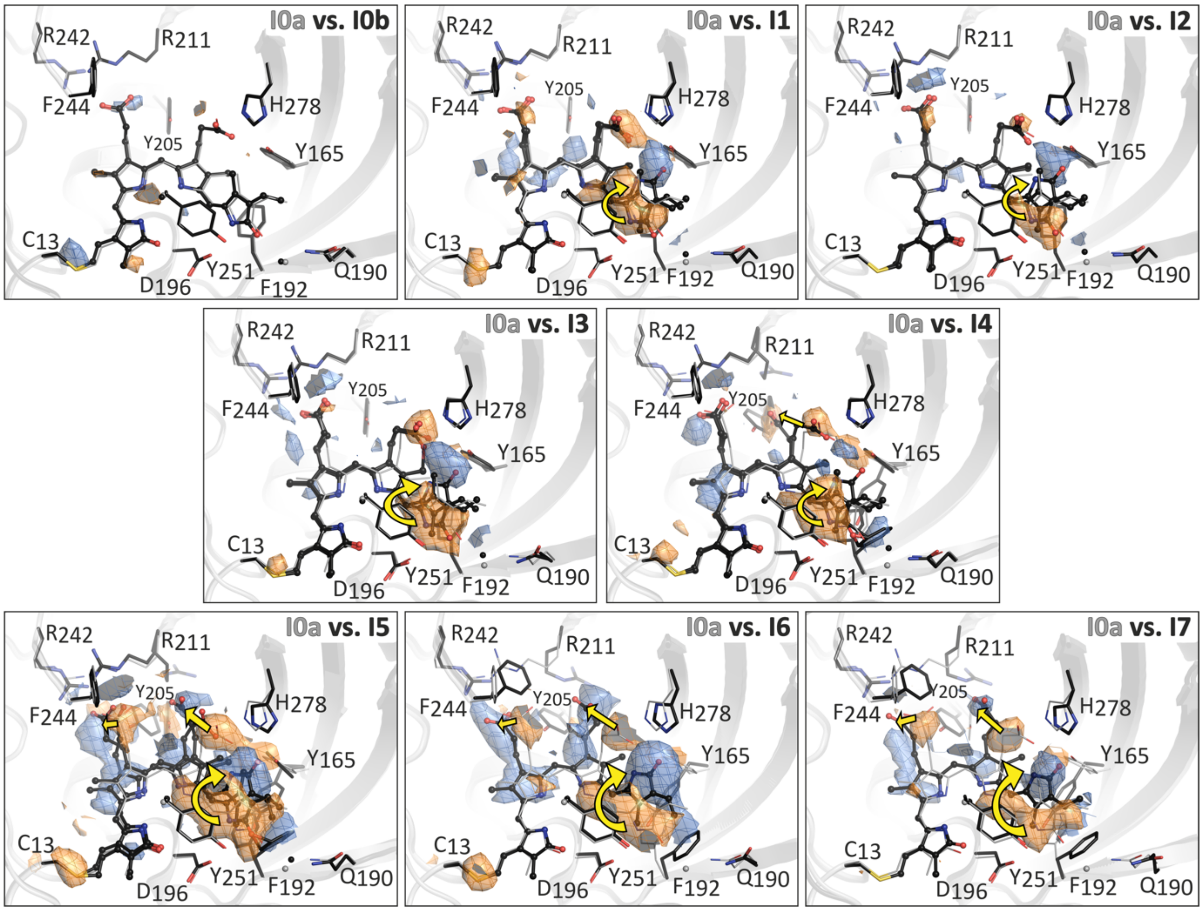

Fig. S4. Time-resolved structural changes of molecule B (MolB) of Agp2-PAiRFP2 visualized in the $Fo^{illuminated\text{-}state}$-$Fo^{dark\text{-}adapted\ state}$ electron density difference map.

The $Fo^{illuminated\text{-}state}$-$Fo^{Pfr\text{-}state}$ electron density difference map of molecule B of Agp2-PAiRFP2 shows structural changes after photoisomerization of the chromophore. Blue colored electron density (contoured at +3 σ) indicates altered features obtained at different pump and delay times, whereas and orange colored electron density (-3 σ) represent features of the initial dark state Pfr. The Fo-Fo electron density maps are contoured at the 3.0 σ level. The protein backbone is shown in cartoon representation. The BV and amino acids side chains of the CBP are represented as balls/sticks and sticks, respectively. In all panels the Pfr state structure I0a, BV and amino acid side chains are colored in light grey. In the Pfr state I0b and intermediate states, structures are shown in black.

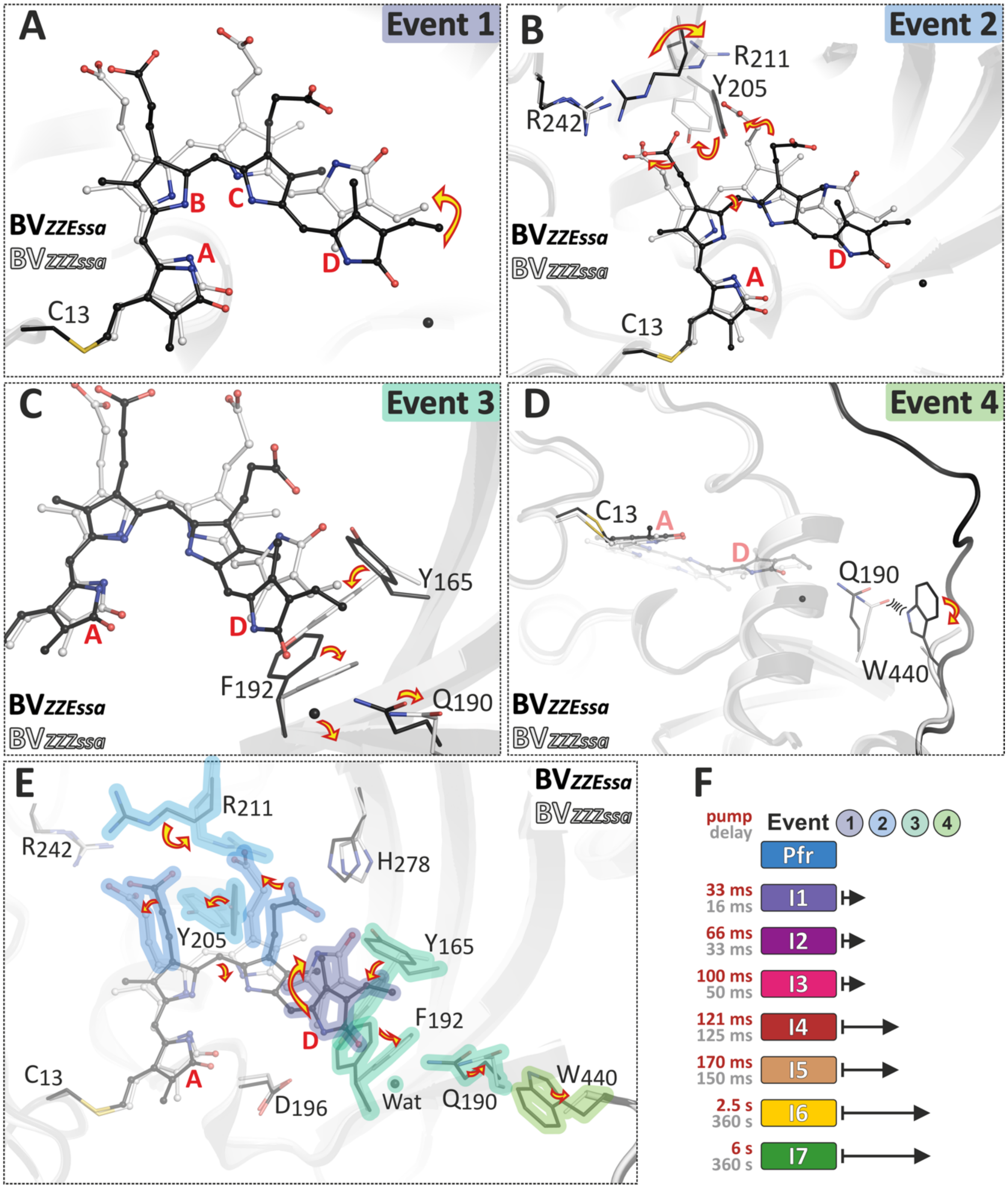

Fig. S5. Sequence of events during the Pfr photoconversion of molecule B (MolB) of Agp2-PAiRFP2. All figures show a superposition of the structures of Pfr I0a (black) and the final I7 (white). (**A**) Event 1 is the isomerization of the chromophore from the *ZZEssa* to the *ZZZssa* configuration. (**B**) Event 2 is the relaxation of the BV skeleton and the *prop-B* and *prop-C* side

chains. This is coupled to structural changes at Tyr205 and Arg211 and accompanied by a reduction of the torsional angle of the *C-D* methine bridge. (**C**) As a result, in event 3, Tyr165 and Phe192 rotate, a water molecule is released from the CBP, and Gln190 moves together with Trp440 from its original position. Structural changes (in form of an unstructuring) of the protein backbone occur in the late part of the photoconversion and represent event 4 (**D**). Figures **E** and **F** show an overview of the structural rearrangements at the chromophore and its binding pocket during the photoconversion. The chromophore is depicted as balls/sticks, the protein backbone as cartoon.

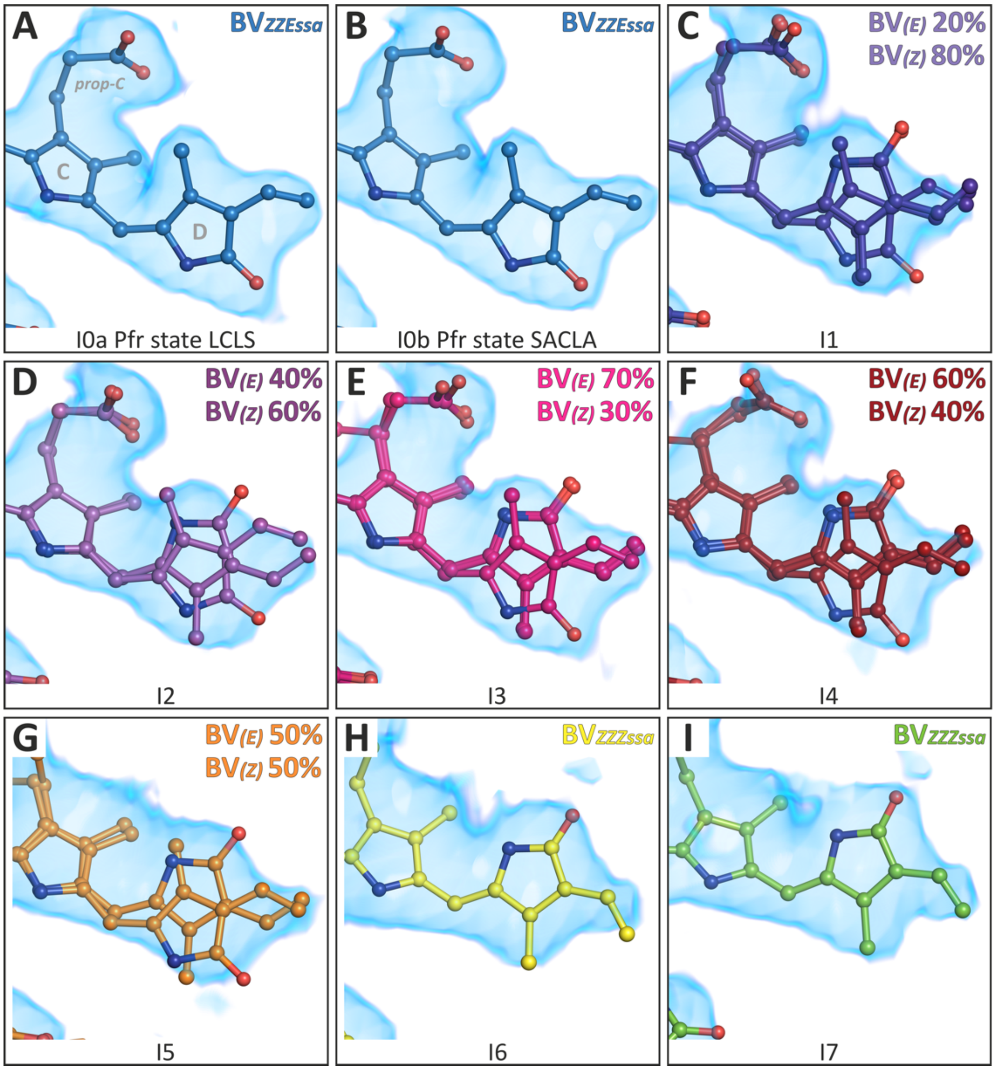

Fig. S6. Group of molecular events 1 – Chromophore isomerization.

Close-up view on ring *D* of the chromophore biliverdin (BV) of Agp2-PAiRFP2 shows the isomerization at the C15-C16 methine bridge as the first step of activation. (**A-B**), BV in Pfr-state exists in *ZZEssa* conformation, (**C-G**), in **I1 - I5**, BV reveals a double conformation and is present in *ZZEssa* and *ZZZssa* conformation in variable percentage (shown in figure), (**H-I**), in **I6** and **I7**, BV completed isomerization and exists in *ZZZssa* conformation only. All figures show the 2*m*Fo-*D*Fc electron densities of the chromophore as volume contoured at 1.0 σ level. BV, the protein

backbone and selected amino acids are depicted in balls and sticks, cartoon and stick representation, respectively.

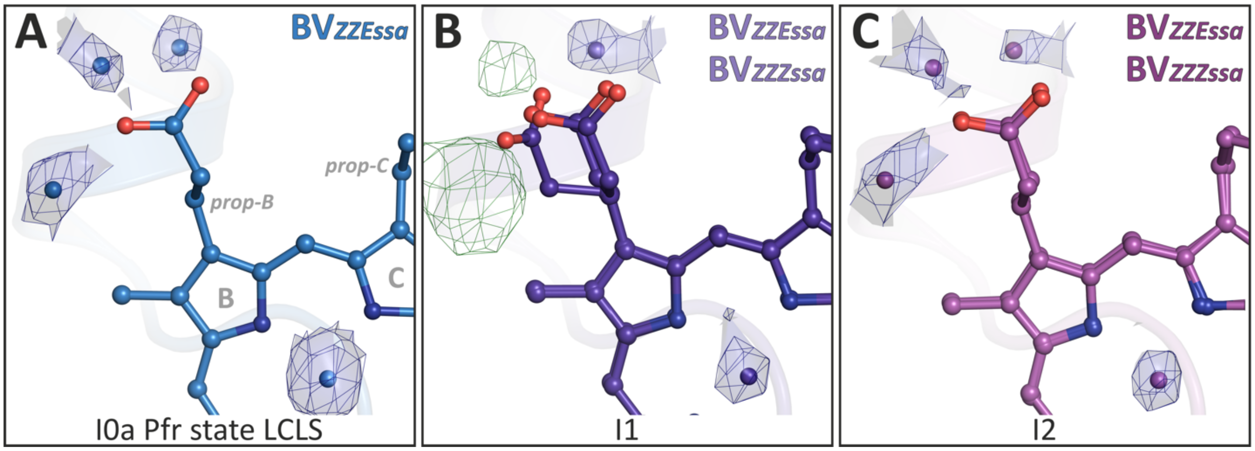

Fig. S7. Group of molecular events 2 – Mobility of water molecules in proximity of *prop-B*. Close-up view on water molecules surrounding *prop-B* of the chromophore of Agp2-PAiRFP2 for the dark state **I0a** (**A**), **I1** (**B**) and **I2** (**C**). **I1** reveals partially the altered conformation of *prop-B* with alternation of the surrounding ordered water molecules. This is clearly indicated by the missing peaks in the 2*m*Fo-*D*Fc and only residual electron density peaks in the *m*Fo-*D*Fc electron density map. In **I2** *prop-B* reverts to its original (initial) conformation and ordered water molecules are structurally interpretable again. All figures show the 2*m*Fo-*D*Fc electron densities of the surrounding water molecules as blue mesh and surface contoured at 1.0 σ level and additionally, in (**B**), the *m*Fo-*D*Fc electron density map as green mesh contoured at 3.0 σ level. BV, the protein backbone and selected water molecules are depicted in balls and sticks, cartoon and sphere representation, respectively.

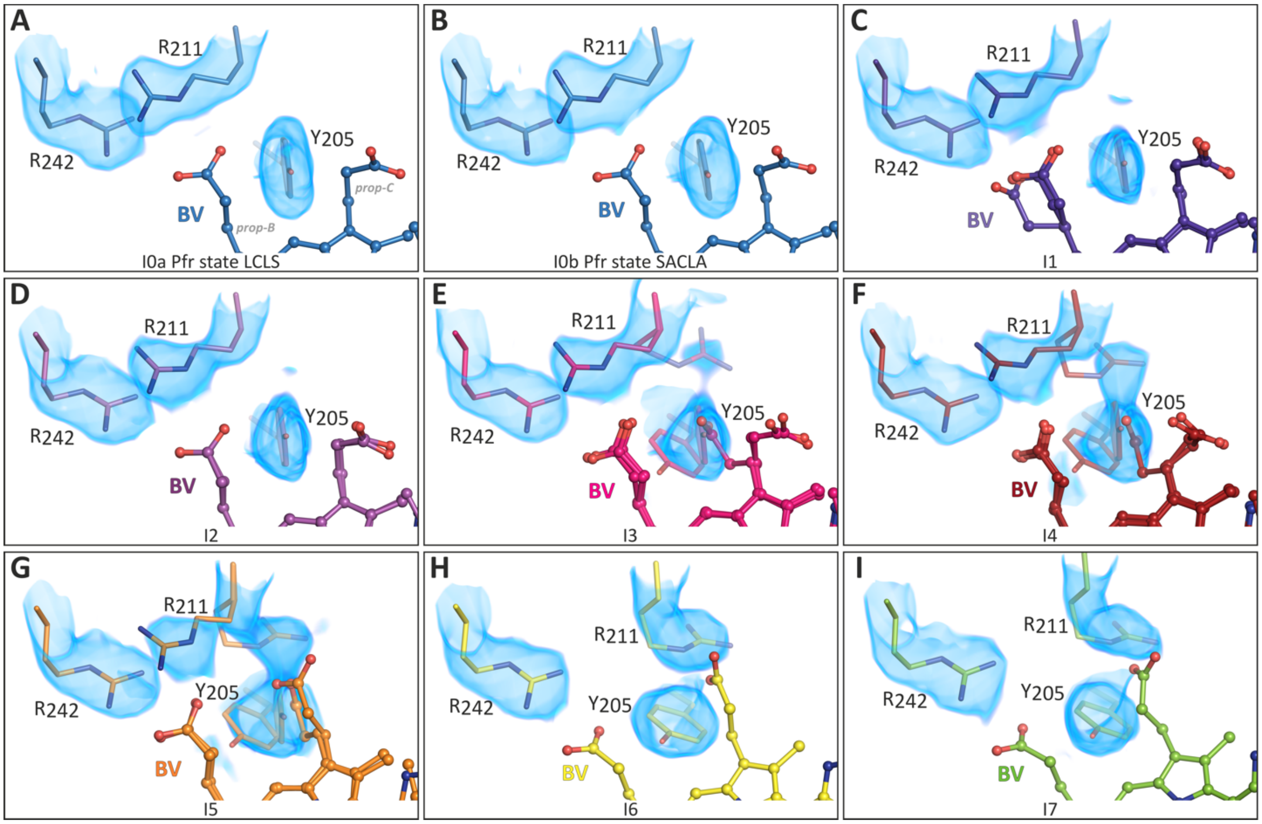

Fig. S8. Group of molecular events 2 – Structural relaxation of the chromophore and adaptations of the hydrogen bond network.

Close-up view on the propionic side chains (*prop-B* and *prop-C*) of the chromophore biliverdin (BV) of Agp2-PAiRFP2 and the residues Tyr205, Arg211 and Arg242. The relaxation of this chromophore region within the chromophore binding pocket proceeds as the second main event of activation. (**A-B**), **I0a** and **I0b** (Pfr state) with biliverdin (BV) and protein indicated in blue; data collected at LCLS and SACLA, respectively; (**C**), **I1** (violet); (**D**), **I2** (purple), (**E**) **I3** (pink), (**F**) **I4** (dark-red), (**G**) **I5** (orange), (**H**) **I6** (yellow), (**I**) **I7** (green). All figures show the 2*m*Fo-*D*Fc electron densities (blue volume) of the chromophore contoured at 1.0 σ level in the different intermediate states. BV, the protein backbone and selected amino acids are depicted in balls and sticks, cartoon and stick representation, respectively.

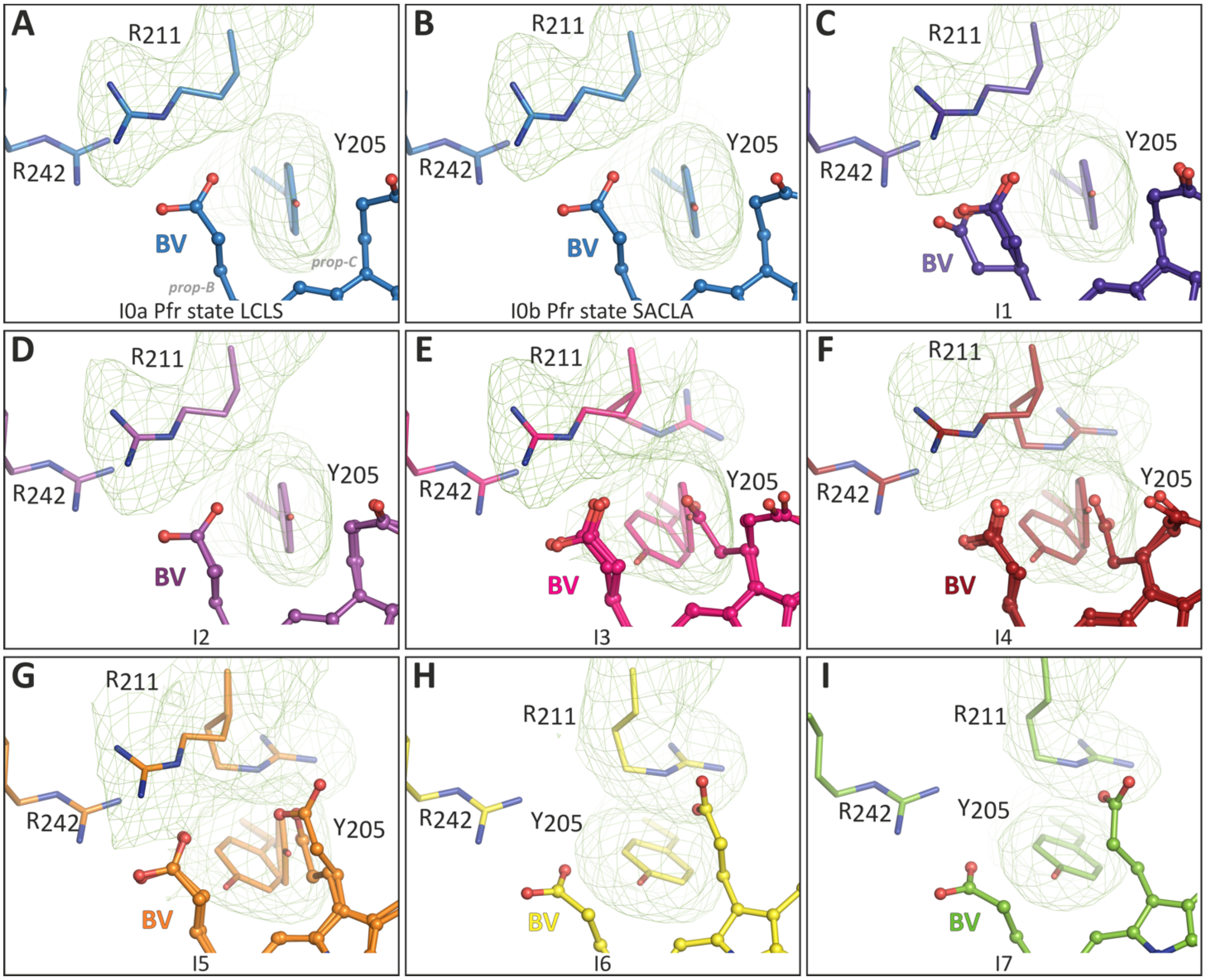

Fig. S9. Group of molecular events 2 – Polder omit map of Tyr205 and Arg211. (**A-I**) show the polder omit maps (*35*) as green meshes contoured at 3.0 σ level for the amino acids Tyr205 and Arg211 undergoing structural changes during relaxation process of *prop-B* and *prop-C*. A double conformation is visible in **I3**. The light triggered structural changes are finalized in **I6** and **I7**. Tyr205 and Arg211 were separately omitted in the calculation of the polder map. BV, the protein backbone and selected amino acids are depicted in balls and sticks, cartoon and stick representation (for the Pfr state **I0a** in blue, **I1** in violet, **I2** in purple, **I3** in pink and **I4** in dark-red, **I5** in orange, **I6** in yellow, **I7** in green).

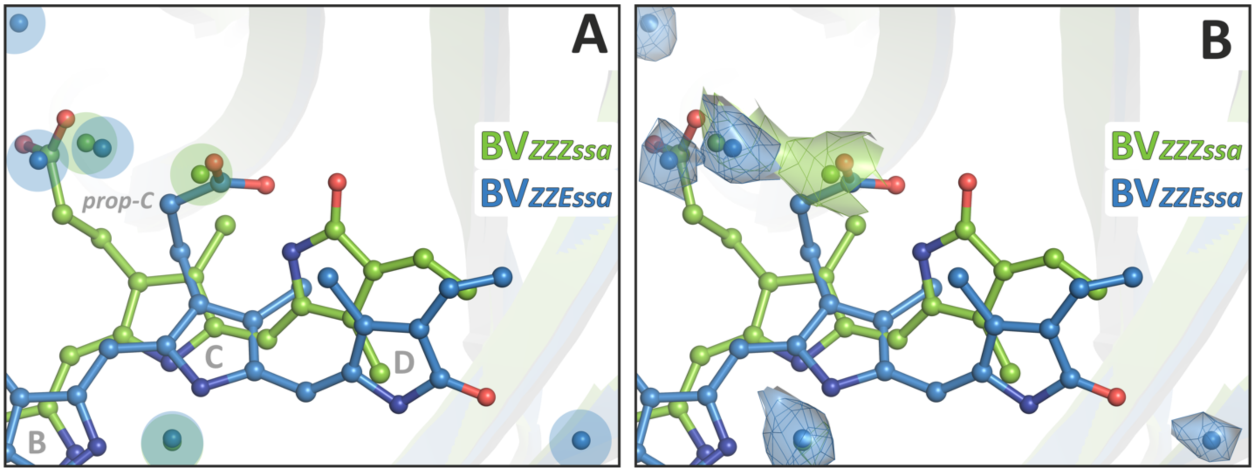

Fig. S10. Changing water network in proximity of *prop-C*.

Superimposition of Agp2-PAiRFP2 structures in Pfr state **I0a** (blue) and the final time frame **I7** (green). The photoinduced chromophore structural changes cause a substantial modification of the water network around *prop-C*. The initial (Pfr) conformation **I0a** of *prop-C* is exchanged with a water molecule in **I7**. Likewise, water molecules present in the Pfr state are exchanged by the light-triggered conformation of *prop-C*. BV, the protein backbone and the water molecules are depicted in balls/sticks, cartoon and sphere representation, respectively. Additionally, water molecules are highlighted (**A**) with colored circles and (**B**) by the 2*m*Fo-*D*Fc electron densities as blue/green mesh and surface contoured at 1.0 σ level.

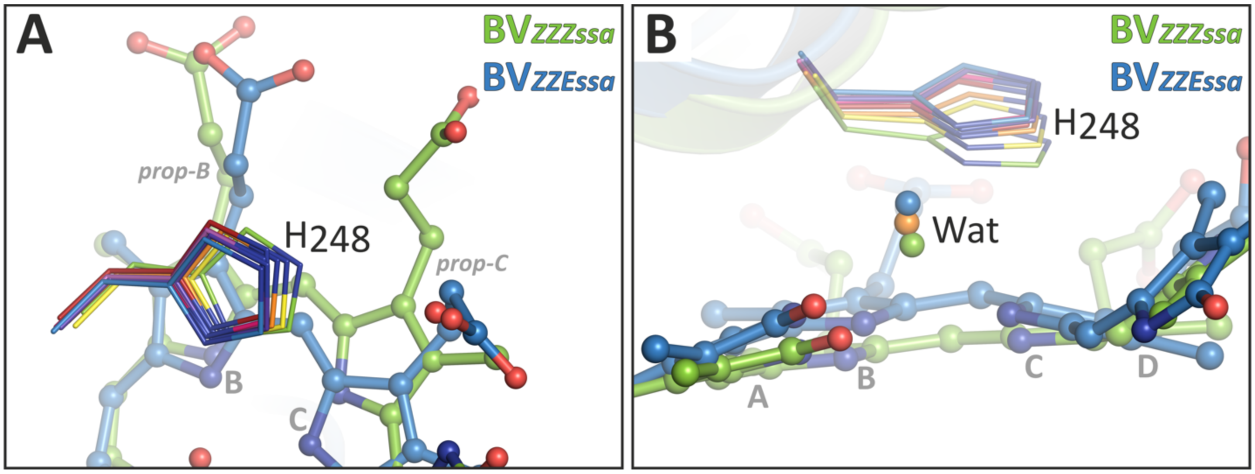

Fig. S11. Movement of conserved His248 during the light-activation of Agp2-PAiRFP2. Superimposition of Agp2-PAiRFP2 structures in **I0a** Pfr state (blue) and in different states (**I1** in violet, **I2** in purple, **I3** in pink, **I4** in dark-red, **I5** in orange, **I6** in yellow and **I7** in green). Photoreaction of BV leads to a small but substantial movement of the highly conserved His248 towards the chromophore position of the Pfr state (**A**). This movement is associated with a change of its hydrogen bond network from in the backbone Ser245, Glu250, Tyr251 and Leu252, the pyrrole water (Wat) and another water molecule (not shown) as well as water mediated to *prop-C* and Ser260 in Pfr state to the backbone Ser245, Glu250, Tyr251 and Leu252, three water molecules (not shown in figure) and temporary to *prop-C* (I3-I6) during the relaxation in **I7**. The differences in distance (Nε of His248) compared to **I0a** are 0.1 Å in **I1**, 0.2 Å in **I2**, 0.3 Å in **I3**, 0.3 in **I4**, 0.4 Å in **I5**, 0.6 Å in **I6** and 0.8 Å in **I7**. (**B**) illustrates a synchronous downward movement of His248 together with the pyrrole water and the rings *B* and *C* including the C-B methine bridge of the chromophore. BV, the protein backbone and the water molecules are depicted in balls/sticks, cartoon and sphere representation, respectively.

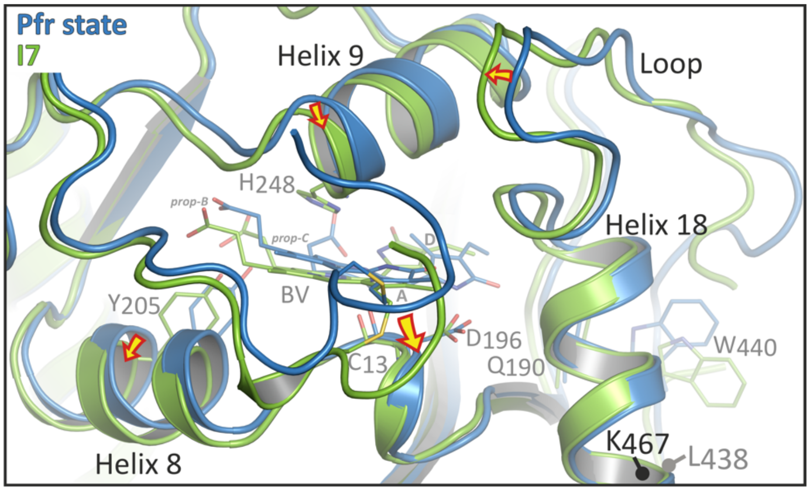

Fig. S12. Structural changes of the protein backbone upon illumination.

Superimposition of Agp2-PAiRFP2 structures in Pfr **I0a** (blue) and **I7** (green) showing movements of the protein backbone. These movements involve helix 8 and 9 as well as helix 18 and the loop from position 438 – 467. The order of the secondary structure numbering was created with ChimeraX (version 1.2) (*94*). BV and selected amino acids, the protein backbone and the water molecules are depicted in line, cartoon and sphere representation, respectively.

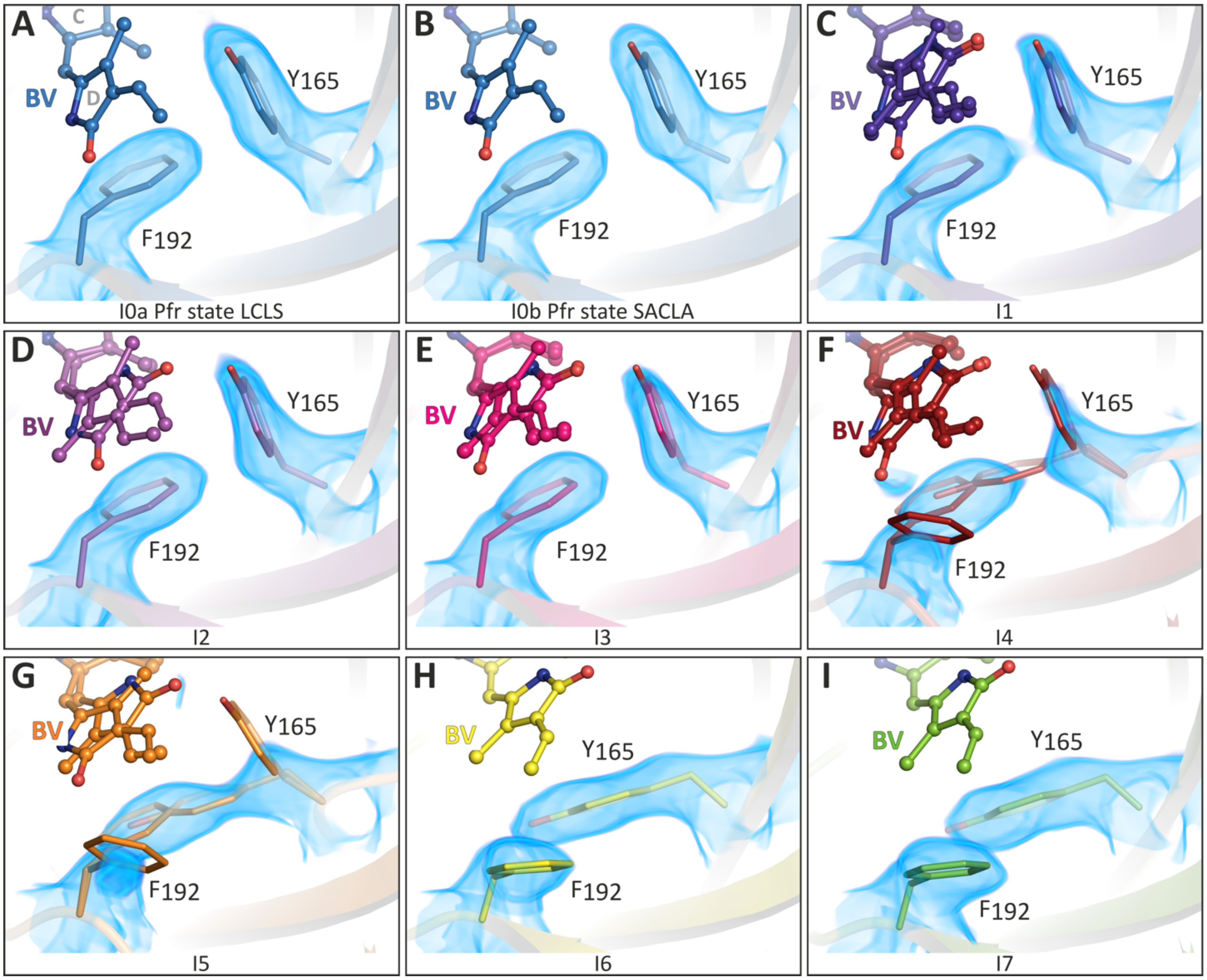

Fig. S13. Group of molecular events 3 – Rearrangements in the chromophore binding pocket.

Close-up view on Tyr165 and Phe192 of the chromophore binding pocket of Agp2-PAiRFP2 in the different time frames. (**A-B**) in Pfr-state **I0a** and **I0b**, Tyr165 is hydrogen bonded to *prop-C* and Leu274, Phe192 points towards Tyr165 forming a hydrophobic pocket around ring *D* of BV, (**C-E**) in **I1-I3**, Tyr165 and Phe192 still display their initial conformation, (**F-G**) in **I4** and **I5**, both amino acids undergo a structural change to Meta-F conformation. Here, the shifted Tyr165 adopts the former position of Phe192, which in turn rotates to the former position of the ring *D* water molecule. In the shifted conformation both amino acids form a hydrophobic cavity stabilizing the

isomerized conformation of ring *D*. Prerequisite for this structural change seems to be the upshift of the rings *C* and *D* of BV that is accompanied by the loss of binding between Tyr165 and *prop-C*. (**H-I**) in **I6** and **I7**, Tyr165 and Phe192 indicate only the light-activated conformation. All figures show the 2*m*Fo-*D*Fc electron densities (blue volumes) of Tyr165 and Phe192 at 1.0 σ level. BV, the protein backbone and selected amino acids are depicted in balls and sticks, cartoon and stick representation, respectively.

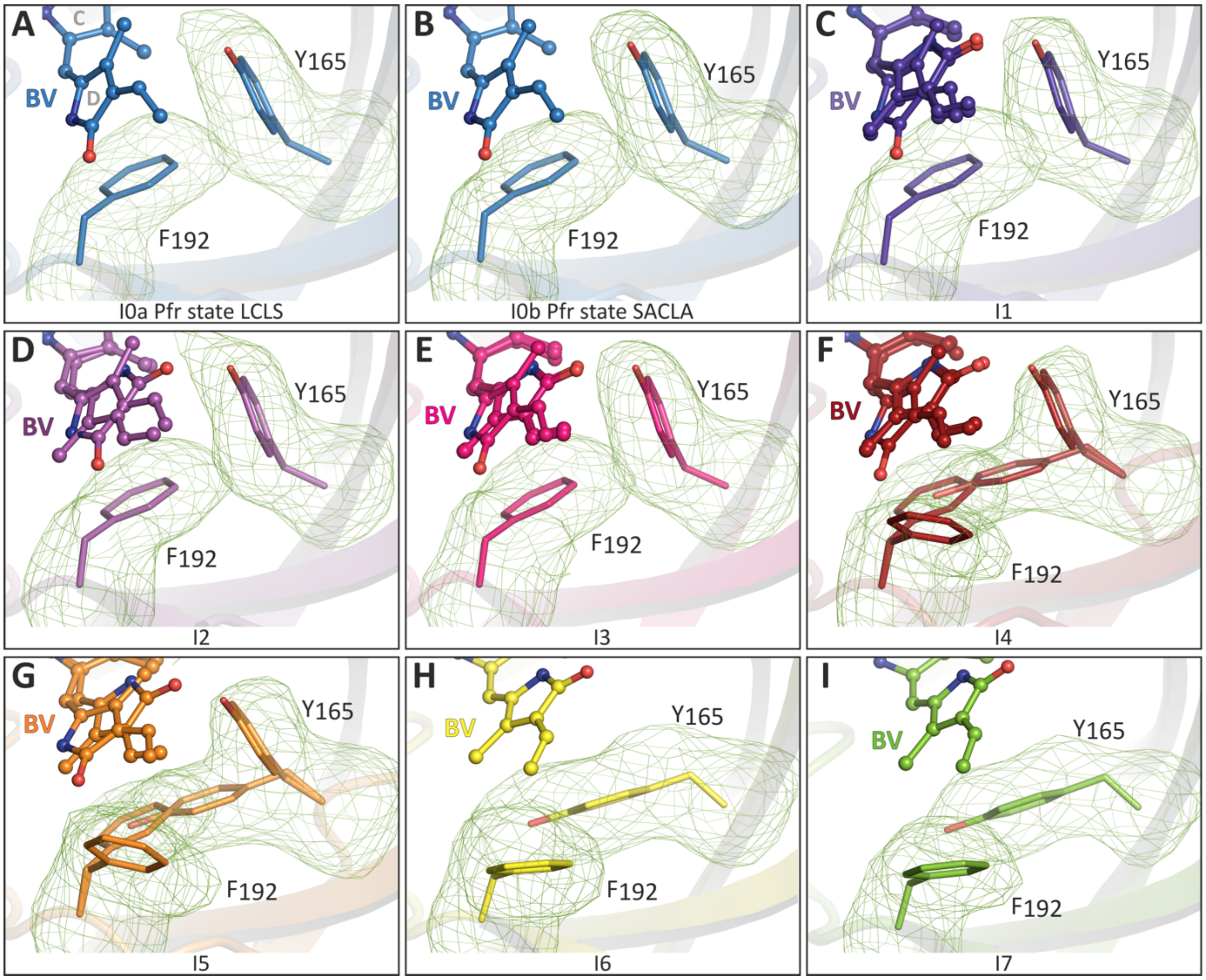

Fig. S14. Group of molecular events 3 - Polder omit maps reveal chronological order.

Close-up view on Tyr165 and Phe192 of the chromophore binding pocket of Agp2-PAiRFP2 in different intermediate states. All figures show the polder omit maps (*35*) (green meshes) of Tyr165 and Phe192 contoured at +3.0 σ level. BV, the protein backbone and selected amino acids are depicted in lines, cartoon and stick representation, respectively. (**A-E**), Tyr165 and Phe192 exist in single Pfr state conformation **I0a**, **I0b** and the **I1**-**I3** time frames; (**F**) in **I4**, Tyr165 and Phe192 reveal for the first time a double conformation; (**G**) in **I5**, the double conformation of both amino acids is more pronounced; (**H**) in **I6** and (**I**) **I7**, Tyr165 and Phe192 display the final light-induced conformation of Meta-F.

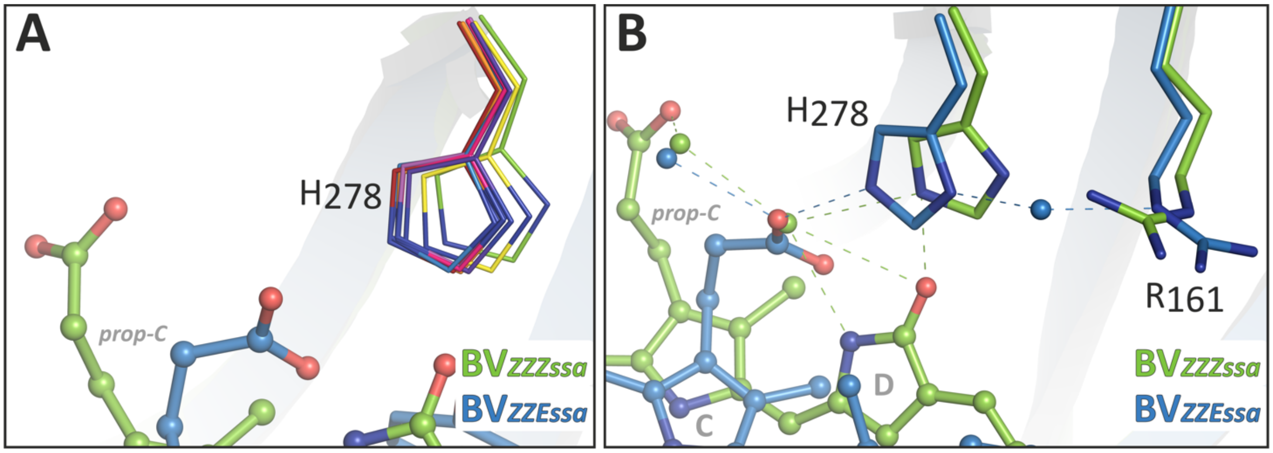

Fig. S15. Movement of conserved His278 during the photoconversion of Agp2-PAiRFP2.

(**A**), Superimposition of Agp2-PAiRFP2 structures in Pfr state (**I0a**, blue) and in different time frames (**I1**, violet; **I2**, purple; **I3**, pink; **I4**, dark-red; **I5**, orange; **I6**, yellow; **I7**, green). The photoinduced reaction cascade of Agp2-PAiRFP2 reveals a small but substantial movement of the highly conserved His278. The differences in distance (Nε of His278) compared to the Pfr-state (**I0a**) are 0.4 Å (**I1**), 0.2 Å (**I2**), 0.2 Å (**I3**), 0.2 Å (**I4**), 0.3 Å (**I5**), 0.8 Å (**I6**), 0.8 Å (**I7**). (**B**), this shift is associated with a change of its hydrogen bond network from *prop-C* and water mediated to Arg161 in **I0a** and to ring *D*, directly to Arg161 and water mediated to *prop-C* in **I7.** BV and the protein backbone are depicted in balls/sticks and cartoon representation, respectively. Water molecules are shown as spheres.

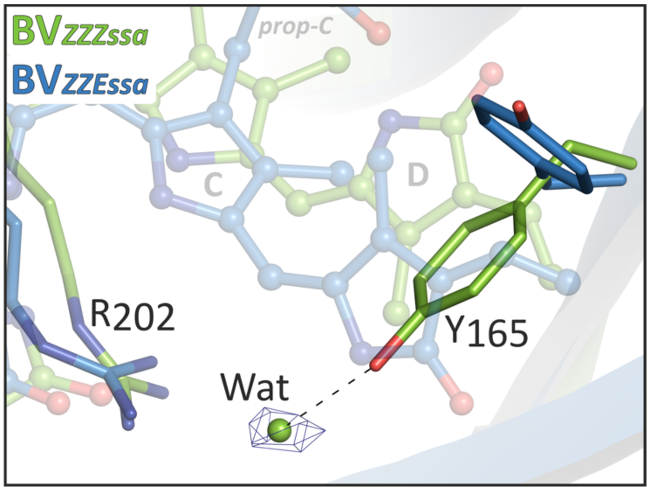

Fig. S16. New water molecule in I7. Superimposition of Agp2-PAiRFP2 structures in Pfr state (**I0a**, blue) and **I7** (green), indicating change of the water network around BV. The final light-induced conformation of Y165 is in hydrogen bond distance to a new water molecule in **I7**. BV, the protein backbone and the water molecules are depicted in balls/stick, cartoon and sphere representation, respectively. The 2*m*Fo-*D*Fc electron density (blue mesh) of the water molecule is contoured at 1.0 σ level for **I7**.

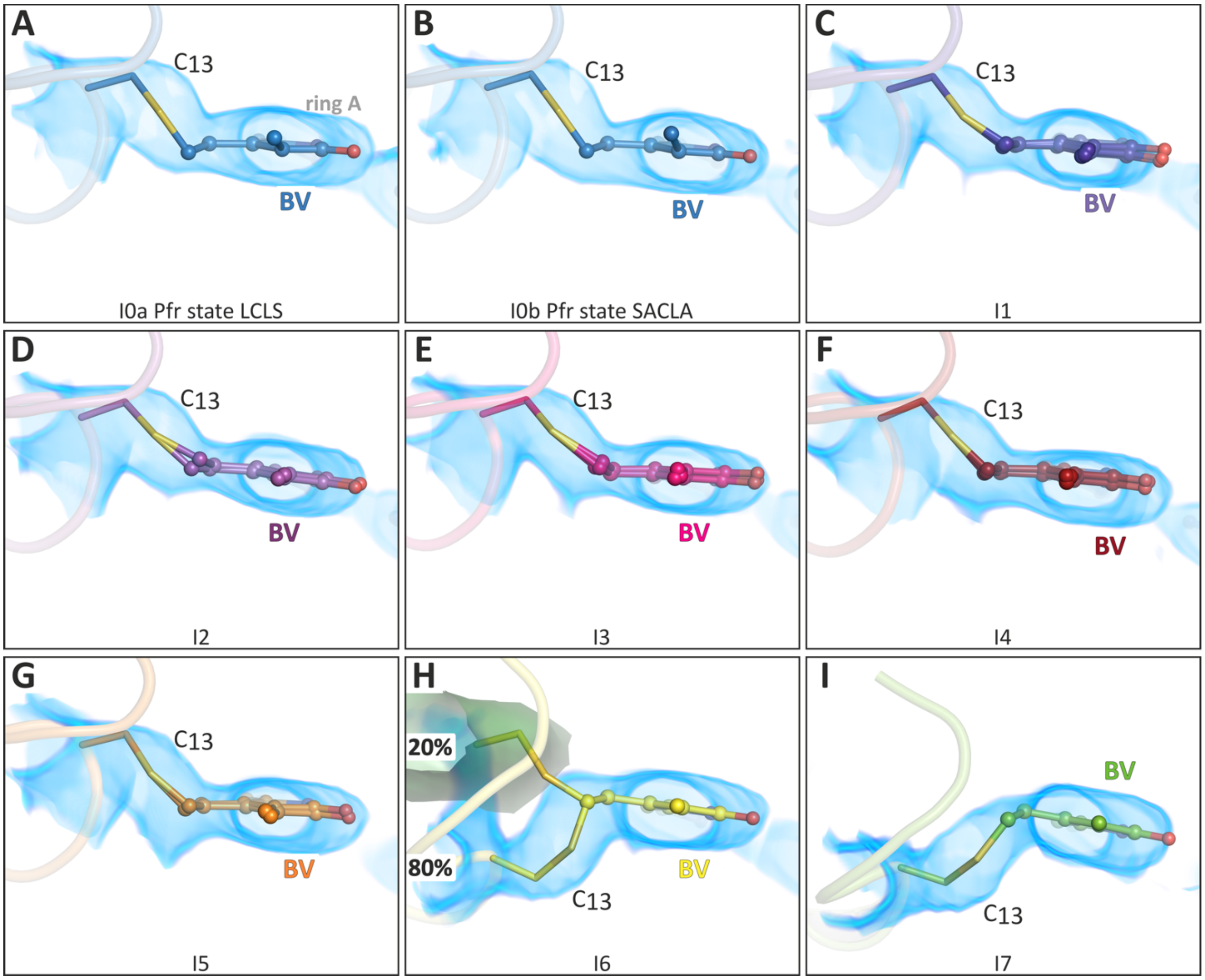

Fig. S17. Group of molecular events 4 – Structural changes of the protein backbone occurring at the N-terminus.

Close-up view on thioether linkage between Cys13 and BV of Agp2-PAiRFP2. (**A-B**), in Pfr state **I0a** and **I0b**, Cys13 is α-facially attached to BV. (**C-G**), no structural changes at the N-terminus are observed in **I1-I5**. A restructuring of the N-terminus is visible in **I6** (**H**) and **I7** (**I**). Here, Cys13 is β-facially bound to BV. The backbone fold of the N-terminus is close to the N-terminus found in Pr of Agp1 (*95*). All figures show the 2*m*Fo-*D*Fc maps of the chromophore and Cys13 contoured at 1.0 σ (blue volume). For **I6** (**H**) the *m*Fo-*D*Fc electron density map is shown as grey-green

surface contoured at 3.0 σ. BV, the protein backbone and selected amino acids are depicted in balls and sticks, cartoon and stick representation, respectively.

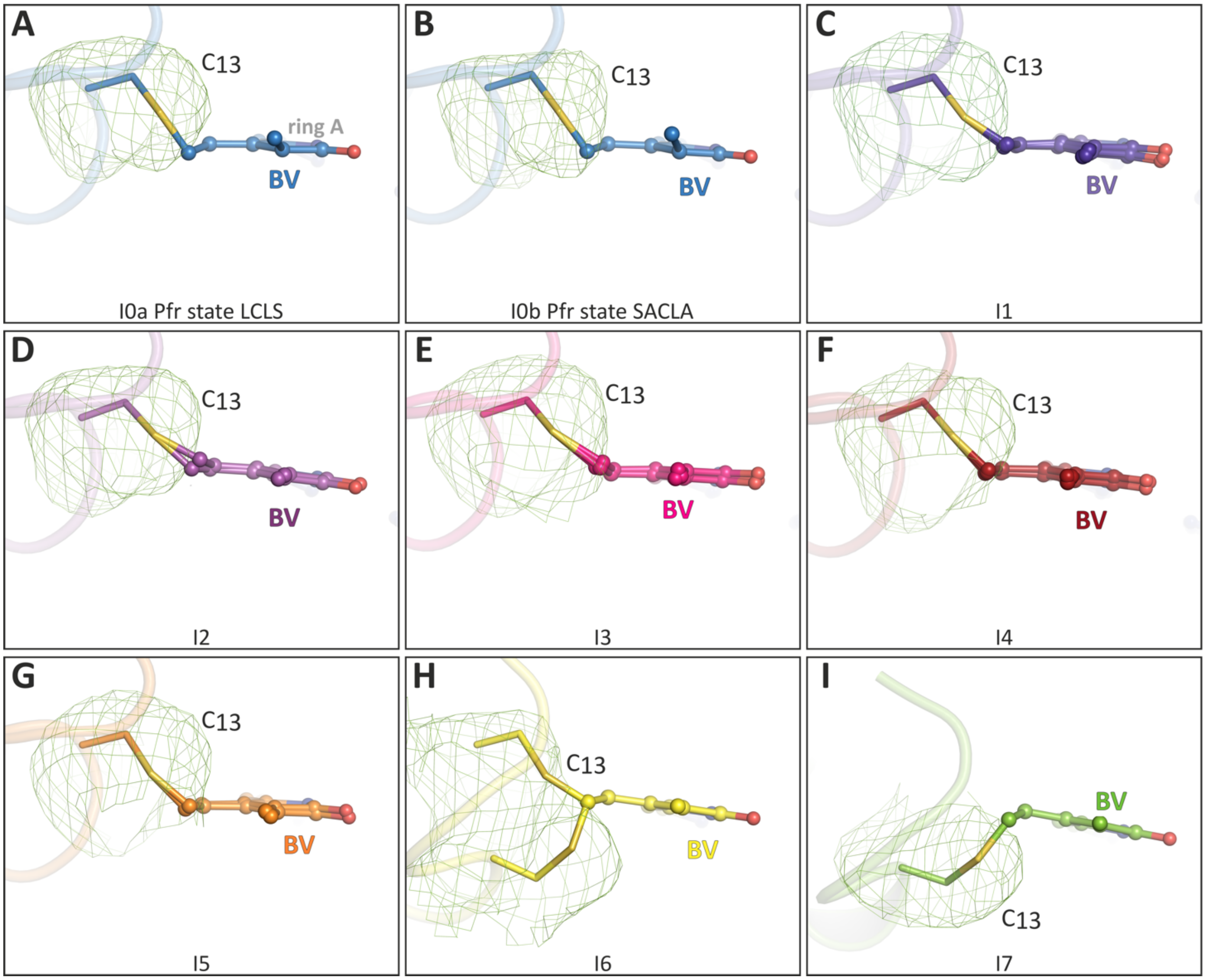

Fig. S18. Group of molecular events 4 – Polder omit maps reveal chronological order of structural changes occurring at the N-terminus.

All figures show the polder omit maps (*35*) (green mesh) for Cys13 contoured at +3.0 σ level in different time frames and reveal the restructuring of the N-terminus as a late event of the photoconversion of Agp2-PAiRFP2. (**A-G**), Cys13 is α-facially attached to the chromophore. A slight rotation of *ring A* is accompanied by the change of the orientation of the thioether linkage of the chromophore. (**H**) and (**I**), in **I6** and **I7** the chromophore is mainly or completely β-facially attached to Cys13, respectively.

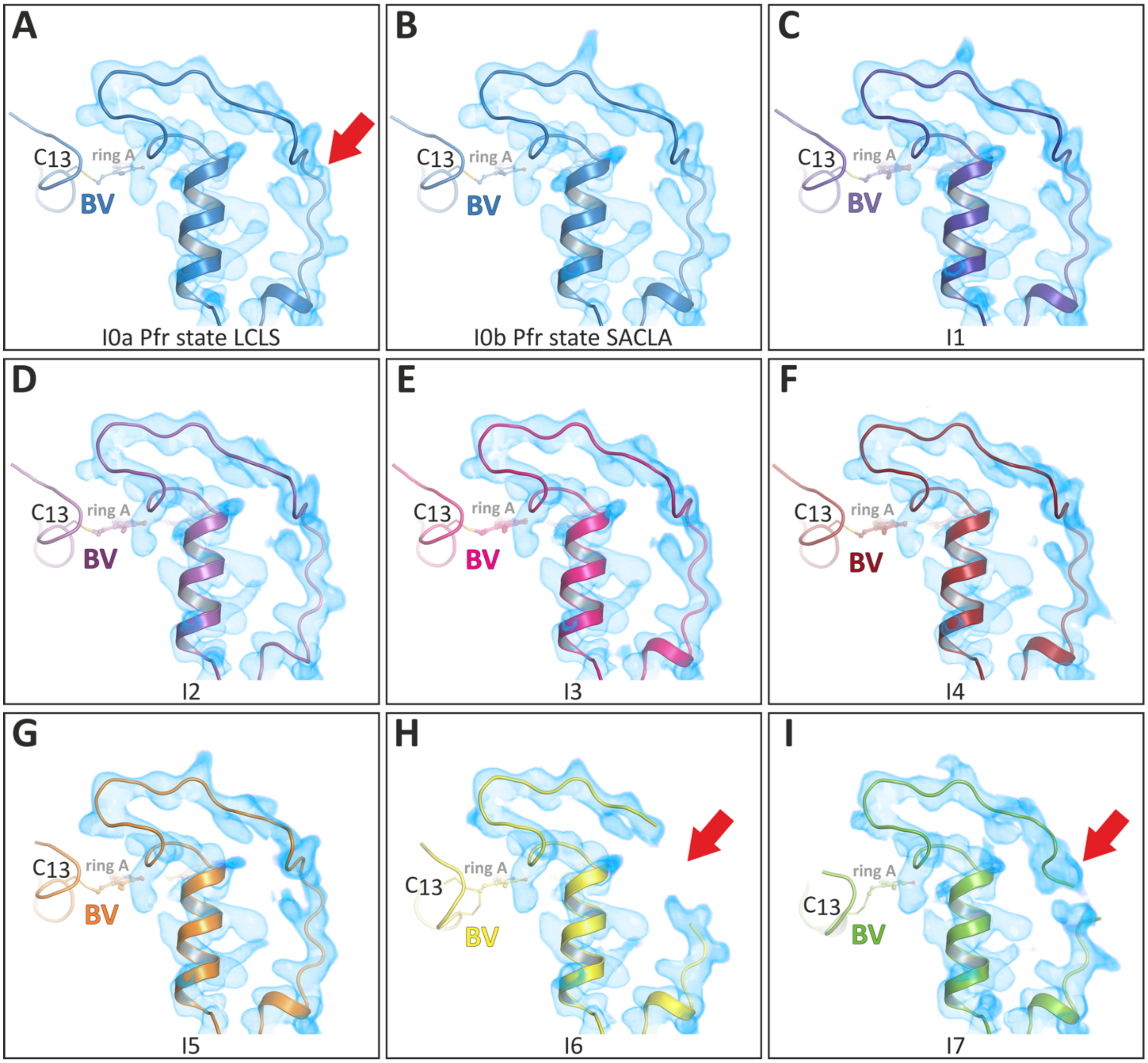

Fig. S19. Group of molecular events 4 – Structural changes of the protein backbone occurring at the PHY tongue.

Close-up view on the tongue region (highlighted in different colors, cartoon representation) of Agp2-PAiRFP2 in different intermediate states. (**A-B**), Pfr states **I0a** and **I0b** (blue) show a structured region containing an α-helix and loops. This region remains unchanged in **I1**-**I5** (**C-G**). An unfolding within a loop region of the tongue starts in **I6** (**H**, yellow). In **I7** (**I**) a refolding of the loop results in a slightly different conformation. Restructuring of the protein backbone is a late

event and requires longer irradiation relaxation times. All figures show the 2*m*Fo-*D*Fc maps (blue volume) of the tongue region (amino acids 431 – 471) contoured at 1.0 σ level. BV shown in the background in balls/sticks representation.

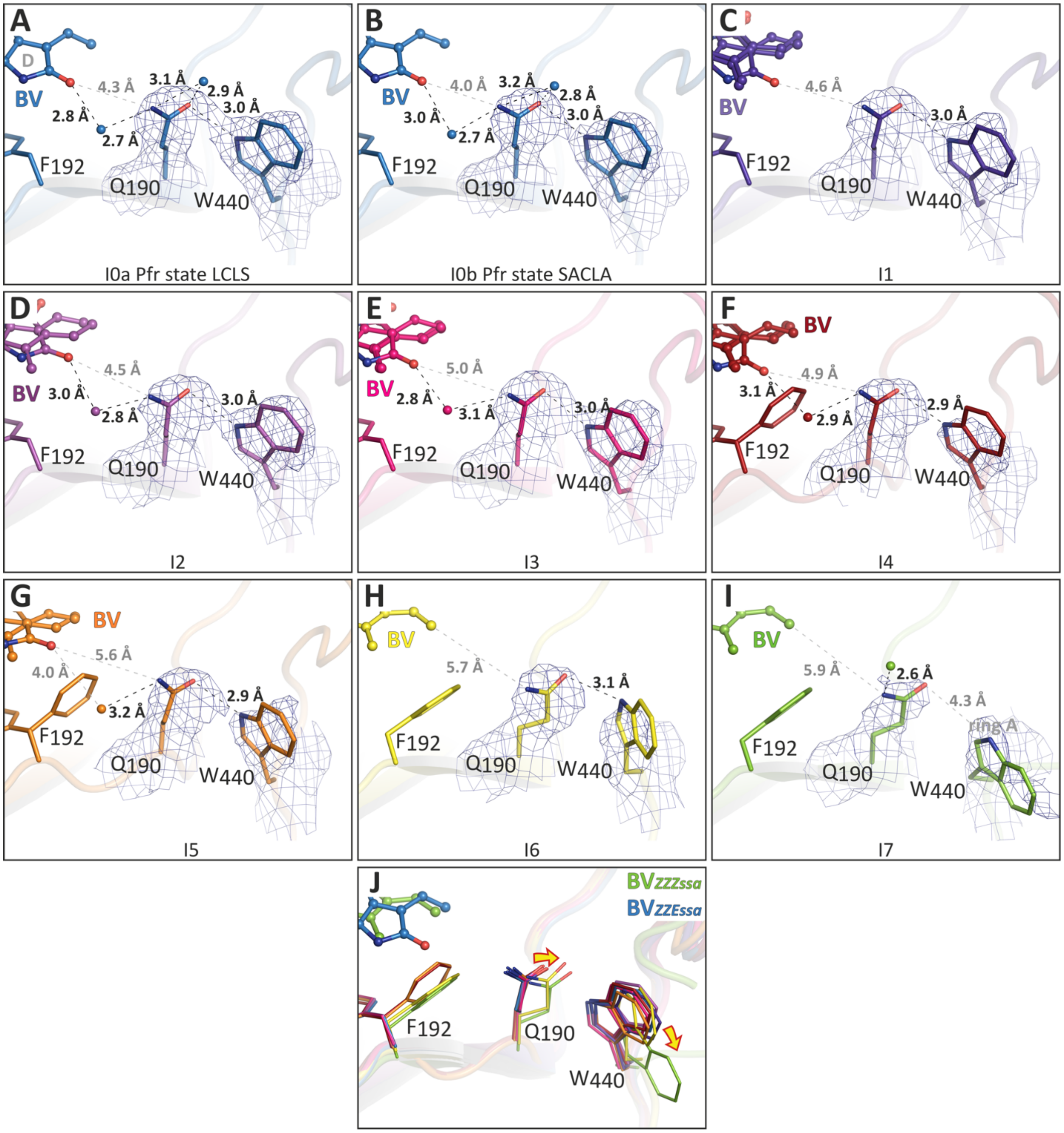

Fig. S20. Time-dependent changes in the interaction of Gln190 and Trp440. Close-up view on Gln190 – Trp440 interaction partners displays a hydrogen bond in Pfr state **I0a** and **I0b** (**A-B**) as well as in the **I1**, **I2**, **I3**, **I4**, **I5** and **I6** time frames (**C-H**). Subsequent to the conformational changes of Phe192, Gln190 performs a shift towards Trp440 in **I6**, which is completed in **I7**. Meanwhile the side chain of Trp440 as well as part of the tongue region shows a high flexibility

thus becomes unstructured in **I6**. In **I7**, Trp440 and the loop part of the tongue region becomes structured into a slightly different fold compared to Pfr. The distance between Gln190 and Trp440 is approximately 4.3 Å, thus out of hydrogen bond interactions. Figures (**A-I**) show the 2*m*Fo-*D*Fc maps contoured at 1.0 σ level (blue meshes). (**J**), superposition of all of these structures to illustrate the stepwise shift of Gln190 and Trp440. BV, the protein backbone and selected amino acids are depicted in balls and sticks, cartoon and stick (line in **J**) representation, respectively; water molecules are shown as spheres.

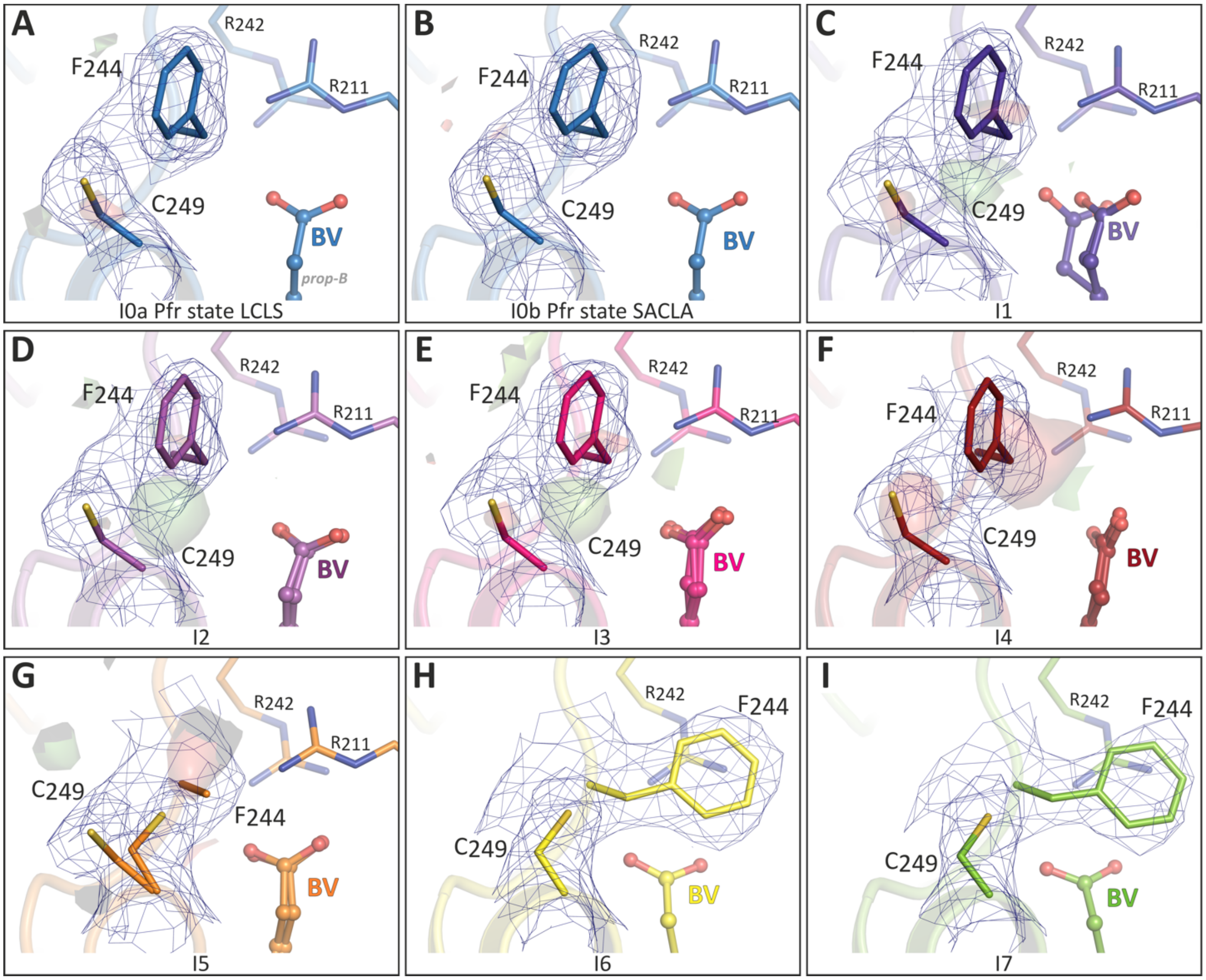

Fig. S21. Time-resolved structural changes of Phe244 and Cys249.

(**A-B**), **I0a** and **I0b** Pfr-state (blue), Phe244 (Agp2-WT Val244) points away from the chromophore BV. Probably due to the bulky side chain of Phe244, Cys249 occupies a different rotamer as in Agp2-WT (*19*). After photoisomerization of BV, in the early time frames **I1**-**I4** (**I1**, violet; **I2**, purple; **I3**, pink; **I4**, dark-red) (**C-F**) single Pfr state conformation can be found, however, additional peaks in the *m*Fo-*D*Fc electron density maps revealing an increased flexibility at Phe244. In **I5** (orange), the Phe244 side chain is highly flexible and Cys249 is able to occupy partially the same conformation as in Agp2-WT. In **I6** and **I7** (**H**, yellow; **I**, green), Phe244 and Cys249 show their final light-induced conformations only. Here, Phe244 is rotated above *prop-B*

of BV. Figures (**A-I**) show the 2*m*Fo-*D*Fc maps (blue mesh) contoured at 1.0 σ level and the *m*Fo-*D*Fc electron densities as green and red surface contoured at 3.0 σ. BV, the protein backbone and selected amino acids are depicted in balls and sticks, cartoon and stick representation, respectively.

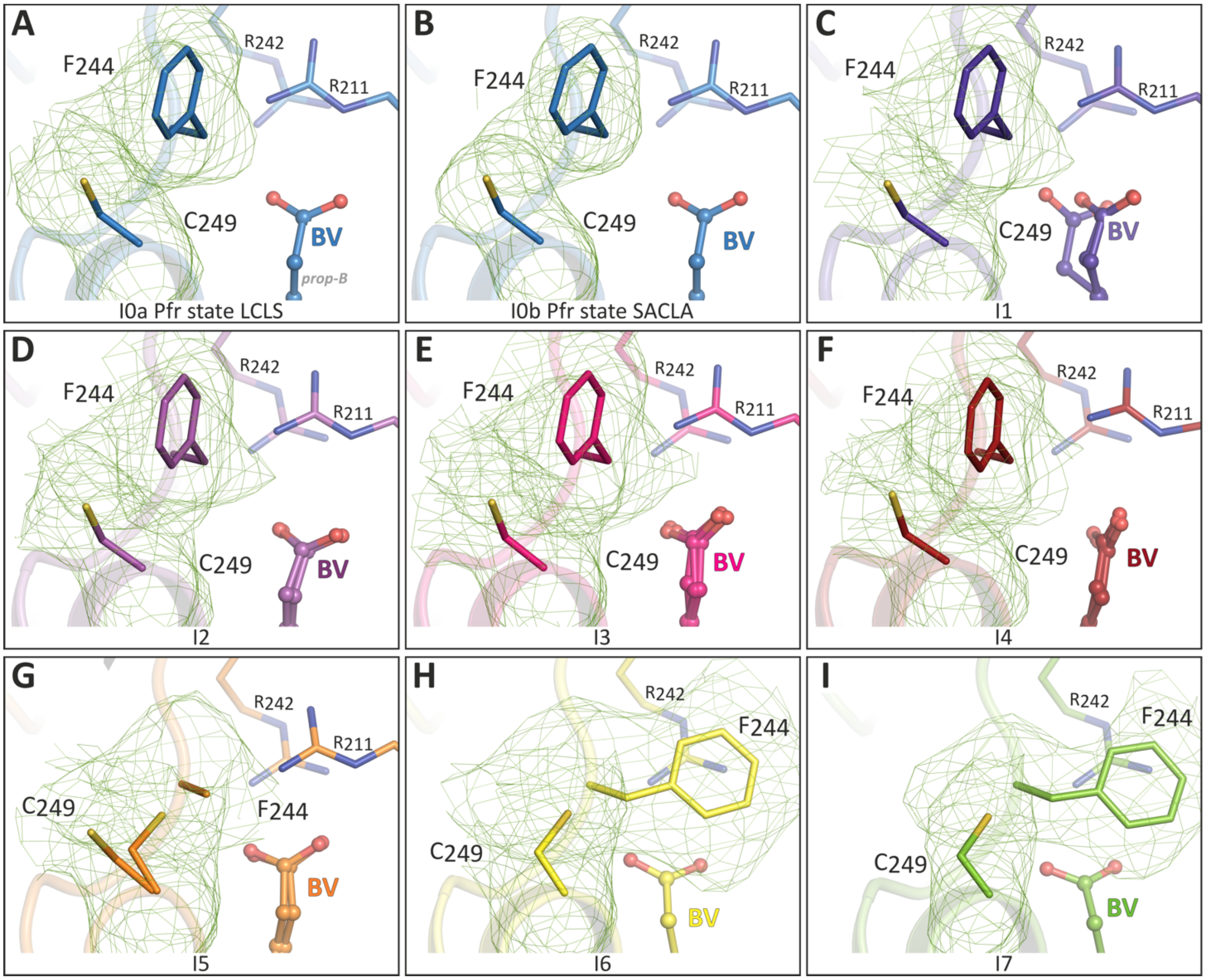

Fig. S22. Chronological ordering of light-induced structural changes at Phe244 and Cys249. In **(A-H)** polder omit maps (*35*) (green meshes, contoured at 3.0 σ level) of both amino acids reveal the conformational switch that starts in **I5**. BV, the protein backbone and selected amino acids are depicted in balls and sticks, cartoon and stick representation, respectively.

Table S1. Overview of illumination conditions used for the different data sets in this study.

Proposal and run #s	XFEL state	Pump time	Delay time between pumping and X-ray probing	Settings used	LED power	Belt speed [mm/s]	Flow rate [µl/min]
LS00 203-237 LS34 263-272	**Pfr I0a**	N/A	N/A	N/A	N/A	300	11
LS00 243-269 316-325	**I6**	2.5 s	360 s	8 mm mask on capillary, ILP3	20 mW unfocused	300	6
LS00 304-315	**I1**	33 ms	16 ms	Free space LED on tape, 5 mm before X-ray, 10 mm spot size on tape	110 mW focused, 3 cm distance, 3 µJ, 10 mm spot size on tape	300	3-4
LS00 326-334 364-373 375-378	**I2**	66 ms	33 ms	Free space LED on tape, ILP1	110mW focused, 3 cm distance, 3 µJ	150	5-6
LS00 380-395	**I3**	100 ms	50 ms	Free space LED on tape, ILP1	110mW focused, 3 cm distance, 3 µJ	100	4
LS34	**I7**	6 s	360 s	32 mm mask on capillary, ILP3	2.7 mJ	300	10
SACLA 62252-62302	**Pfr I0b**	N/A	N/A	N/A	N/A	300	14
SACLA 62310-62381	**I5**	170 ms	150 ms	Free space LED on tape, ILP2	17 mm spot size, 100% intensity	100	12
SACLA 62647-62691	**I4**	121 ms	125 ms	17 mm spot size on tape 17.5 mm before X-ray, ILP2	770 nm, 100% intensity	140	9

Table S2. Data collection and refinement statistics of Pfr states **I0a/I0b** and the **I1** data sets.

	**Agp2-PAiRFP2**<br>**Pfr state – I0a**<br>(PDB-ID 8RJM)	**Agp2-PAiRFP2**<br>**Pfr state – I0b**<br>(PDB-ID 8RJN)	**Agp2-PAiRFP2**<br>**I1**<br>(PDB-ID 8RJO)
**Illumination conditions**			
Illumination time	-	-	33 ms
Delay time	-	-	16 ms
λ (nm)	-	-	780
**Data collection**	LCLS, MFX	SACLA, BL2 EH3	LCLS, MFX
(wavelength)	λ = 1.30129 Å	λ = 1.24026 Å	λ = 1.30042 Å
Space group	$P6_322$	$P6_322$	$P6_322$
Cell dimensions			
a, *b*, *c* (Å)	184.98 184.98 182.54	184.19 184.19 181.63	185.09 185.09 182.28
α, β, γ (°)	90.0, 90.0, 120.0	90.0, 90.0, 120.0	90.0, 90.0, 120.0
Crystal lattices merged	45,331	14,431	12,794
Resolution (Å)	25.75 – 2.15<br>(2.21 – 2.15)*	92.27 – 2.20<br>(2.26 – 2.20)*	25.41– 2.54<br>(2.61 – 2.54)*
$<I/\sigma(I)>$	39.0 (0.7)	16.4 (1.3)	24.3 (0.3)
cc1/2	89.7 (7.9)	95.7 (4.3)	84.1 (6.2)
Completeness (%)	99.9 (100.0)	100.0 (100.0)	99.8 (100.0)
Multiplicity	309.0 (30.0)	100.8 (61.6)	98.4 (20.9)
**Refinement**			
No. Reflections	99,757	92,007	61,030
R_{work} / R_{free} (%)	20.4 / 21.7	21.3 / 24.1	20.6 / 23.6
No. atoms			
Protein	7,560	7,511	7,518
Ligand/ion	110	101	235
Water	304	216	203
B-factors			
Protein	62.4	64.0	72.5
Ligand/ion	67.3	66.2	79.4
Water	61.8	59.4	62.6
R.m.s† deviations			
Bond lengths (Å)	0.014	0.014	0.014
Bond angles (°)	1.58	1.59	1.59
Ramachandran plot‡			
Favoured (%)	98.9	98.1	97.6

Allowed (%)	1.1	1.9	2.4
Outlier (%)	0.0	0.0	0.0

[*] highest resolution shell is shown in parenthesis; [†] R.m.s, root mean square; [‡] Ramachandran plot calculated by MolProbity (*66*)

Table S3. Overview of main group events 1 and 2.

	**Group 1**	**Group 2**<br>BV relaxation		Amino acid rearrangements		
Data set Agp2-PAiRFP2	***Ring D***	***prop-C***	***prop-B***	**Y205**	**R211**	**R242**
Pfr state I0a LCLS	*ZZEssa*; H-bond to Wat5, D196, Y251	H-bond to Wat12, Y165, H278	H-bond to Wat40, Wat42, Wat52, R211	H-bond to Wat40, A201, R202, S264	H-bond to *prop-B*, Wat37, R242, N243, S262	H-bond to Wat52, Wat95, G25, R211, L236, A239
Pfr state I0b SACLA	*ZZEssa*; H-bond to Wat4, D196, Y251	H-bond to Wat7, Y165, H248, H278	H-bond to Wat32, Wat34, Wat42, R211	H-bond to Wat32, A201, R202, S264	H-bond to *prop-B*, Wat30, Wat61, R242, N243, S262	H-bond to Wat42, Wat322, G25, R211, L236, A239
I1	Small upshift, partially isomerized (20:80): *ZZEssa*: H-bond to D196; *ZZZssa*: Y165	H-bond to Wat163, H278	Partial shift (60:40); H-bond to Wat110, R211, R242	H-bond to A201, S264	H-bond to *prop-B*, Wat164, R242, S262	H-bond to *prop-B*, Wat84, R211, A239
I2	Small upshift, partially isomerized (40:60): *ZZEssa*: H-bond to Wat105, Y251; *ZZZssa*: Y165	H-bond to W26, Y165, H278	H-bond to Wat21, Wat23, Y205, R211	H-bond to *prop-B*, A201, R202, S264	H-bond to *prop-C*, Wat104, R242, S262	H-bond to Wat21, Wat22, I18, G25, R211, A239

I3	Small upshift, partially isomerized (70:30): *ZZEssa*: H-bond to Wat47, D196, Y251; *ZZZssa*: Y165	Partial shift (85:15); H-bond to Wat79, Y165, Y205, H248, H278	R211, R242	Partial shift (70:30); *prop-C*, A201, S264	Partial shift (70:30); H-bond to *prop-B*, Wat25, N208, T209, R242, S262, S264	H-bond to *prop-B*, I18, G25, R211, A239
I4	Small upshift, partially isomerized (60:40): *ZZEssa*: H-bond to Wat3, D196, Y251; *ZZZssa*: Y165	Partial shift (80:20); H-bond to Y205, H248, S262, H278	H-bond to Wat200, Y205, R211	Partial shift (60:40); H-bond to *prop-C*, *prop-B*, A201, A203, S264	Partial shift (60:40); H-bond to *prop-B*, Wat144, N208, T209, R242, S262, S264	H-bond to *prop-B*, Wat88, G25, R211, A239
I5	Upshift, partially isomerized (50:50): *ZZEssa*: H-bond to D196, Y251; *ZZZssa*: Y165	Upshift, partial shift (50:50); H-bond to Wat22, Y205, S262	H-bond to Y205, R211, R242	Partial shift (50:50); H-bond to *prop-B*, *prop-C*, A201, S264	Partial shift (50:50); H-bond to *prop-B*, *prop-C*, N208, R242, S262, S264	H-bond to *prop-B*, Wat57, G25, R211, A239
I6	Upshift, *ZZZssa*; H-bond to Wat1, H278	Upshift, H-bond to Wat36, R211, S262	H-bond to Wat136, Y205, R242, S245	H-bond to *prop-B*, A201, A203, S264	H-bond to *prop-C*, N208, S262, S264	H-bond to *prop-B*, Wat112, I18, A239
I7	Upshift, *ZZZssa*;	Upshift, H-bond to Wat66,	H-bond to Y205,	H-bond to *prop-B*, A201,	H-bond to *prop-C*, N208,	H-bond to *prop-B*,

H-bond to Wat1, H278	R211, S260, S262	R242, S245	R202, S264	S262, S264	Wat18, G25, A239

Table S4. Overview of main group events 3 and 4.

	**Group 3**			**Group 4**	
Data set Agp2-PAiRFP2	**Y165**	**F192**	**Q190**	**N-term**	**PHY tongue**
Pfr state I0a LCLS	H-bond to *prop-C*, L274	H-bond to G173	H-bond to Wat5, Wat74, V175, F187, W440, P459	α-facial C13 attachment	Completely resolved
Pfr state I0b SACLA	H-bond to *prop-C*, L274	H-bond to G173	H-bond to Wat4, Wat56, V175, F187, W440, P459	α-facial C13 attachment	Completely resolved
I1	H-bond to ring *D*, *prop-C*, L274	H-bond to G173	H-bond to V175, Phe187 L188, W440, P459	α-facial C13 attachment	Completely resolved
I2	H-bond to *prop-C*, ring *D*, L274	H-bond to G173	H-bond to Wat105, V175, F187, L188, W440, P459	α -facial C13 attachment	Completely resolved
I3	H-bond to *prop-C*, ring *D*, L274	H-bond to G173	H-bond to Wat47, V175, F187, W440, P459	α-facial C13 attachment	Completely resolved
I4	Partial shift (60:40); H-bond to ring *D*, L274	Partial shift (60:40); H-bond to G173	H-bond to Wat3, V175, F187, L188, W440, P459	α-facial C13 attachment	Completely resolved
I5	Partial shift (50:50); H-bond to ring *D*, L274	Partial shift (50:50); H-bond to G173	H-bond to Wat33, V175, F187, L188, W440, P459	α-facial C13 attachment	Completely resolved
I6	H-bond to L274	H-bond to G173	H-bond to V175, F187, L188, W440	Double conformation (20:80);	Unresolved (441 – 445); W440 shifted

				α- and β-facial C13 attachment	
I7	H-bond to Wat14, L274	H-bond to G173	H-bond to Wat77, V175, F187, L188	β-facial C13 attachment	Almost completely resolved (442), restructured, shift of W440

Table S5. Data collection and refinement statistics of the **I2**, **I3** and **I4** data sets.

	**Agp2-PAiRFP2 I2** (PDB-ID 8RJP)	**Agp2-PAiRFP2 I3** (PDB-ID 8RJQ)	**Agp2-PAiRFP2 I4** (PDB-ID 8RJR)
**Illumination conditions**			
Illumination time	66 ms	100 ms	121 ms
Delay time	33 ms	50 ms	125 ms
λ (nm)	780	780	770
**Data collection**	LCLS, MFX	LCLS, MFX	SACLA, BL2 EH3
(wavelength)	λ = 1.30087 Å	λ = 1.30096 Å	λ = 1.24052 Å
Space group	$P6_322$	$P6_322$	$P6_322$
Cell dimensions			
a, *b*, *c* (Å)	184.73 184.73 181.70	184.64 184.64 182.17	183.76 183.76 180.32
α, β, γ (°)	90.0, 90.0, 120.0	90.0, 90.0, 120.0	90.0, 90.0, 120.0
Crystal lattices merged	23,308	49,031	23,055
Resolution (Å)	25.64 – 2.43 (2.49 – 2.43)*	26.03 – 2.40 (2.46 – 2.40)*	90.32 – 2.30 (2.36 – 2.30)*
$<I/\sigma(I)>$	25.8 (0.4)	31.9 (0.4)	13.5 (1.6)
cc1/2	90.1 (2.7)	92.4 (3.4)	98.4 (1.2)
Completeness (%)	99.9 (100.0)	99.9 (100.0)	100.0 (100.0)
Multiplicity	220.4 (16.9)	533.4 (24.5)	506.8 (243.9)
**Refinement**			
No. Reflections	69,042	68,403	79,712
R_{work} / R_{free} (%)	20.2 / 23.4	20.3 / 22.1	24.9 / 28.5
No. atoms			
Protein	7,551	7,465	7,617
Ligand/ion	270	282	244
Water	220	115	167
B-factors			
Protein	68.9	72.1	63.9
Ligand/ion	77.9	82.3	72.5
Water	63.0	64.1	51.7
R.m.s[†] deviations			
Bond lengths (Å)	0.020	0.014	0.013
Bond angles (°)	1.64	1.60	1.61
Ramachandran plot[‡]			
Favoured (%)	98.4	98.3	97.7

Allowed (%)	1.6	1.8	2.3
Outlier (%)	0.0	0.0	0.0

* highest resolution shell is shown in parenthesis; † R.m.s, root mean square; ‡ Ramachandran plot calculated by MolProbity (*66*)

Table S6. Data collection and refinement statistics the **I5**, **I6** and **I7** data sets.

	**Agp2-PAiRFP2 I5** (PDB-ID 8RJS)	**Agp2-PAiRFP2 I6** (PDB-ID 8RJT)	**Agp2-PAiRFP2 I7** (PDB-ID 8RJU)
**Illumination conditions**			
Illumination time	170 ms	2.5 s	6 s
Delay time	150 ms	360 s	360 s
λ (nm)	770	780	780
**Data collection**	SACLA, BL2 EH3	LCLS, MFX	LCLS, MFX
(wavelength)	λ = 1.24028 Å	λ = 1.30040 Å	λ = 1.31196 Å
Space group	$P6_322$	$P6_322$	$P6_322$
Cell dimensions			
a, *b*, *c* (Å)	184.10 184.10 180.96	185.20 185.20 182.01	182.90 182.90 180.84
α, β, γ (°)	90.0, 90.0, 120.0	90.0, 90.0, 120.0	90.0, 90.0, 120.0
Images	22,367	22,216	38,485
Resolution (Å)	92.22 – 2.43 (2.49 – 2.43)*	25.44 – 2.49 (2.56 – 2.49)*	45.77 – 2.80 (2.87 – 2.80)*
$<I/\sigma(I)>$	19.8 (1.2)	26.4 (0.2)	17.6 (1.2)
cc1/2	97.8 (1.4)	88.2 (1.2)	92.8 (20.5)
Completeness (%)	100.0 (100.0)	99.9 (100.0)	100.0 (100.0)
Multiplicity	194.9 (119.0)	177.1 (18.2)	890.5 (147.8)
**Refinement**			
No. Reflections	68,292	64,685	44,427
R_{work} / R_{free} (%)	21.0 / 24.8	20.9 / 25.0	27.6 / 32.1
No. atoms			
Protein	7,514	7,411	7,373
Ligand/ion	198	127	157
Water	88	128	337
B-factors			
Protein	76.5	77.6	61.5
Ligand/ion	87.8	96.0	87.3
Water	63.6	65.3	47.6
R.m.s† deviations			
Bond lengths (Å)	0.014	0.014	0.014
Bond angles (°)	1.60	1.60	1.63
Ramachandran plot‡			

Favoured (%)	97.6	97.8	96.4
Allowed (%)	2.4	2.2	3.4
Outlier (%)	0.0	0.0	0.0

[*] highest resolution shell is shown in parenthesis; [†] R.m.s, root mean square; [‡] Ramachandran plot calculated by MolProbity (*66*)

Table S7. Overview of the *m*Fo-*D*Fc difference peak intensity and hydrogen bond interaction partners of the pyrrole water (see also fig. S11B).

Agp2-PAiRFP2 in different time frames	*m*Fo-*D*Fc (e/Å^3)*	*m*Fo-*D*Fc rmsd*	H-bond interaction partners
**Pfr state I0a LCLS**	0.65	13.10	N atoms of rings *A*, *B*, and *C* of BV, His248
**Pfr state I0b SACLA**	0.56	12.20	N atoms of rings *A*, *B*, and *C* of BV, His248
**I1**	0.44	9.60	N atoms of rings *A*, *B*, and *C* of BV, O atom of ring *A*, His248
**I2**	0.46	10.00	N atoms of rings *A*, *B*, and *C* of BV, O atom of ring *A*, His248
**I3**	0.45	10.20	N atoms of rings *A*, *B*, and *C* of BV, O atom of ring *A*, His248
**I4**	0.36	7.40	N atoms of rings *A*, *B*, and *C* of BV, O atom of ring *A*, His248
**I5**	0.34	8.10	N atoms of rings *A* and *C* of BV, O atom of ring *A*, His248
**I6**	0.37	8.30	N atoms of rings *A*, *B*, and *C* of BV, O atom of ring *A*, His248
**I7**	0.41	6.13	N atoms of rings *A*, *B*, and *C* of BV, O atom of ring *A*, His248

* Peaks were calculated with the full structure in the absence of the pyrrole water

Movie S1.

Movie of the sequence of structural changes after light activation of the phytochrome Agp2-PAiRFP2, starting from Pr (I0) via the intermediate times frames I1-I6 to the final time frame I7 (length: 19s).
